# Niche partitioning by photosynthetic plankton as a driver of CO_2_-fixation across the oligotrophic South Pacific Subtropical Ocean

**DOI:** 10.1038/s41396-021-01072-z

**Published:** 2021-08-19

**Authors:** Julia Duerschlag, Wiebke Mohr, Timothy G. Ferdelman, Julie LaRoche, Dhwani Desai, Peter L. Croot, Daniela Voß, Oliver Zielinski, Gaute Lavik, Sten Littmann, Clara Martínez-Pérez, Bernhard Tschitschko, Nina Bartlau, Helena Osterholz, Thorsten Dittmar, Marcel M. M. Kuypers

**Affiliations:** 1 Max Planck Institute for Marine Microbiology, Bremen, Germany; 2 Department of Biology, Dalhousie University, Halifax, NS Canada; 3 iCRAG (Irish Centre for Research in Applied Geoscience), Earth and Ocean Sciences, School of Natural Sciences and the Ryan Institute, National University of Ireland Galway, Galway, Ireland; 4 Institute for Chemistry and Biology of the Marine Environment, University of Oldenburg, Oldenburg, Germany; 5 Marine Perception Research Group, German Research Center for Artificial Intelligence (DFKI), Oldenburg, Germany; 6 Helmholtz Institute for Functional Marine Biodiversity (HIFMB), University of Oldenburg, Oldenburg, Germany; 7 Present Address: Department of Geophysical Sciences, University of Chicago, Chicago, IL USA; 8 Present Address: Institute for Environmental Engineering, Department of Civil, Environmental and Geomatic Engineering, Eidgenössische Technische Hochschule (ETH) Zürich, Zurich, Switzerland; 9 Present Address: Leibniz Institute for Baltic Sea Research Warnemünde, Rostock, Germany

**Keywords:** Biooceanography, Biogeochemistry

## Abstract

Oligotrophic ocean gyre ecosystems may be expanding due to rising global temperatures [1–5]. Models predicting carbon flow through these changing ecosystems require accurate descriptions of phytoplankton communities and their metabolic activities [6]. We therefore measured distributions and activities of cyanobacteria and small photosynthetic eukaryotes throughout the euphotic zone on a zonal transect through the South Pacific Ocean, focusing on the ultraoligotrophic waters of the South Pacific Gyre (SPG). Bulk rates of CO_2_ fixation were low (0.1 µmol C l^−1^ d^−1^) but pervasive throughout both the surface mixed-layer (upper 150 m), as well as the deep chlorophyll *a* maximum of the core SPG. Chloroplast 16S rRNA metabarcoding, and single-cell ^13^CO_2_ uptake experiments demonstrated niche differentiation among the small eukaryotes and picocyanobacteria. *Prochlorococcus* abundances, activity, and growth were more closely associated with the rims of the gyre. Small, fast-growing, photosynthetic eukaryotes, likely related to the Pelagophyceae, characterized the deep chlorophyll *a* maximum. In contrast, a slower growing population of photosynthetic eukaryotes, likely comprised of Dictyochophyceae and Chrysophyceae, dominated the mixed layer that contributed 65–88% of the areal CO_2_ fixation within the core SPG. Small photosynthetic eukaryotes may thus play an underappreciated role in CO_2_ fixation in the surface mixed-layer waters of ultraoligotrophic ecosystems.

## Introduction

Ocean gyre ecosystems are characterized by high-light intensities, extremely low nutrient concentrations and deep chlorophyll *a* maxima (chl *a* max) that are registered as very low chl *a* regions in satellite data. Marine oligotrophic regions, which are dominated by subtropical gyre ecosystems, contribute 19 to 23% to the total fixation of CO_2_ into marine biomass (i.e., net primary production) [7, 8]. Subtropical oceans are also regions of net CO_2_ drawdown from the atmosphere into the ocean [9]. The fixation of CO_2_ via photosynthesis into biomass and the net export of some fraction of this biomass into sub-euphotic zone waters plays an important role in this CO_2_ drawdown. A number of studies have suggested that ocean gyres are expanding [1–5], leading to decreased chl *a* and associated CO_2_ fixation within gyre cores [3, 5, 10]. The relationship of CO_2_ fixation rates to surface chl *a* content in ocean gyres is, however, complicated; CO_2_ fixation in gyre systems is often uncoupled from chl *a* contents [5, 11, 12]. A recent niche-partitioning model [6] also suggests that surface water warming increases pico-phytoplankton biomass in lower latitude ecosystem, which contrasts with Earth System models that employ simplified, indeterminate ecosystem descriptions for predicting productivity changes in oligotrophic regions [6]. Furthermore, global ocean circulation model results suggest that net dissolved organic carbon (DOC) export is responsible for ~50% of the fixed C export out of oligotrophic gyre ecosytems, and that this DOC export is strongly correlated with ecosystem structure in the form of picoplankton community composition [13].

Fully understanding photosynthetic phytoplankton diversity and activity is thus critical for predicting how biomass responds to changing environmental conditions, e.g., warming and expansion of ocean gyres. A substantial fraction of CO_2_ fixation in oligotrophic gyre ecosystems is carried out by small photosynthetic plankton (<1–5 µm), which includes picocyanobacteria and small photosynthetic eukaryotes [14–16]. Picocyanobacteria belonging to the genus *Prochlorococcus* are the most abundant phytoplankton in the oligotrophic subtropical and tropical open ocean systems [17–19]. Small photosynthetic eukaryotes are not as abundant as *Prochlorococcus*. They can significantly contribute, however, to biomass and CO_2_ fixation rates in oligotrophic and mesotrophic marine ecosystems due to their relatively large size [20–22]. How these key groups of CO_2_ fixing organisms respond to changing temperature and nutrient regimes is a topic of intense research [6, 23].

Due to its size and remoteness, the South Pacific Gyre (SPG) ecosystem may provide a glimpse into how the photosynthetic community and corresponding rates of CO_2_ fixation evolves in expanding and deepening ocean gyres (both future and past, i.e., Cenozoic Ocean). The SPG is the largest ocean gyre ecosystem, and is the central feature of the oligotrophic South Pacific that covers nearly 10% of the oceans’ surface area [24, 25]. It has the clearest waters with depleted chl *a* concentrations of less than 0.03 µg l^−1^ in surface waters [2, 26]. Although the SPG is often referred to as being ultraoligotrophic or an “oceanic desert” [26], the few studies where CO_2_ fixation in the euphotic zone of the SPG has been experimentally measured show that there are low but detectable rates of CO_2_ fixation (~0.08–0.17 µmol C l^−1^ d^−1^) [27–29]. Bacterial diversity and distribution closely correlate with the clear, oligotrophic and high-light intensity conditions specific to the SPG [30], suggesting that core SPG waters harbor a distinct microbial community.

**Fig. 1:**
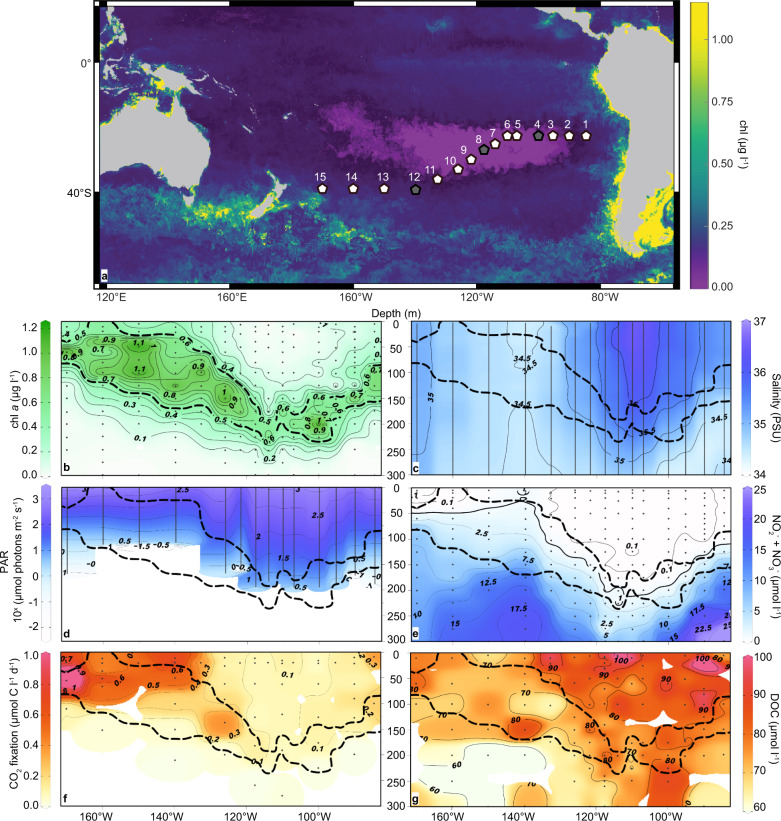
Distribution of physical, chemical, and biological properties along the SO245 UltraPac transect. **a** Cruise track of the SO245 UltraPac Expediton with monthly composite MODIS image (January 2016) [97] of sea surface chlorophyll (chl) concentration. Pentagons indicate Stations sampled. Stations marked in gray indicate sites where single-cell uptake analyses were performed. Transect distributions of (**b**) chl *a*, (**c**) salinity; (**d**) photosynthetically active radiation (PAR), (**e**) nitrate plus nitrite; (**f**) volumetric rates of CO_2_ fixation into biomass and (**g**) DOC concentrations [35]. Black dots mark sampling or measurement depths, and the thick dashed lines represent the 0.5 µg l^−1^ chl *a* isolines and delineate the chl *a* max.

Our goal was to elucidate the structure of the key groups of the photosynthetic plankton community, in particular picocyanobacteria and small photosynthetic eukaryotes, and link these groups to quantitative estimates of CO_2_ fixation on a basin-wide transect through the core of the oligotrophic SPG and into the adjacent mesotrophic Southwest Pacific (SWP) (Fig. 1a). We measured rates of photosynthetic CO_2_ fixation and the abundance of pico-phytoplankton, including *Prochlorococcus* and small eukaryotes (1–5 µm), through the upper mixed layer into the deep chl *a* max. We analyzed the chloroplast 16S rRNA gene amplicon reads to assess the diversity and distribution of photosynthetic eukaryotes through the cross-basin transect and compared these with 16S rRNA and 18S rRNA gene reads from select metagenomes. Moreover, we examined the single-cell CO_2_ fixation rates, growth and the contribution of photosynthetic small eukaryotes and *Prochlorococcus* to the CO_2_ fixation using nano-scale secondary ion mass spectroscopy (nanoSIMS). Our study shows that the niche-partitioning among small photosynthetic eukaryotes and picocyanobacteria drives low, but similar rates of CO_2_ fixation within the deep chl *a* maximum and throughout the overlying 150 m deep, clear, ultraoligotrophic water column of the core SPG.

## Materials and methods

### Sampling, hydrography, chemistry, and underwater light field

Sampling was carried out at 15 stations during the *RV Sonne* “UltraPac” cruise (SO245) from Antofagasta, Chile (17 December 2015) to Wellington, New Zealand (28 January 2016) (Fig. 1a; Supplementary File 2). Temperature, salinity, dissolved oxygen and density were examined using a CTD (Sea-Bird Scientific SBE 911plus probe, WA, USA) attached to a Carousel Water Sampler (SBE 32) (ref. [31]). Dissolved inorganic phosphorus (PO_4_^3−^), dissolved inorganic nitrogen (DIN as the sum of NO_2_^−^ and NO_3_^−^) and silicate (Si) concentrations were measured with a QuAAtro39 autoanalyzer (Seal Analytical, Germany) [32]. Chlorophyll *a* (chl a) samples were filtered (1 l) onto 25 mm GF/F filters (Whatman). Chl *a* was extracted in 90% acetone and concentrations were measured fluorometrically [33, 34] (Turner Designs fluorometer, CA, USA, calibrated against chl *a* standard, Sigma-Aldrich, Germany). DOC and total dissolved nitrogen (TDN) data were taken from Osterholz et al. (ref. [35]).

A HyperPro II profiling system (Sea-Bird Scientific, WA, USA, formerly Satlantic) with hyperspectral irradiance and one hyperspectral radiance sensor was used to collect hyperspectral underwater light field data in free-falling mode [36]. A second hyperspectral irradiance sensor was mounted on the research vessel for matching above-water irradiance reference measurements. Profiles were conducted at each station depending on sea and weather conditions, with deployments at 50 m away from the ship to avoid ship shadow.

### CO_2_ fixation experiments

Rates of CO_2_ fixation into biomass were determined at six depths over the upper water column (<300 m) at Stations 1 (only two depths), 2, 4, 6, 8, 10, 12, 14, and 15. CO_2_ fixation rates were determined with triplicate 24 h incubations of 5 l seawater samples amended with ^13^C-labeled dissolved inorganic carbon (DI^13^C) (NaH^13^CO_3_, ≥98% at% ^13^C, Sigma-Aldrich) [37]. We define CO_2_ fixation as the uptake of inorganic carbon (CO_2_ + HCO_3_^−^ + CO_3_^2−^) into biomass over a 24 h, dawn-to-dawn incubation period. These experiments yield instantaneous rates that should lie close to rates of net primary productivity [38], i.e., the net rate of inorganic carbon incorporation into biomass due to photosynthesis. (See also Supplementary Information for more details.) Filter foils were selected based on Station 1 light spectral intensity profiles to bracket light intensity and spectral conditions in three on-deck incubators: Ocean Blue (Lee filter 724, 36.2% transmission) for the upper two depths, Special Medium Blue (Lee filter 363, 4.2% transmission) for the middle two depths, and Tokyo Blue (Lee filter 071, 1% transmission) for the lower two depths within and immediately below the chl *a* max (Figs. S1, S2; Supplementary File 2). At the end of the incubation period water was filtered for determination of ^13^C into particulate organic carbon by isotope ratio mass spectrometry. Prior to filtration, subsamples (100–200 ml) of incubated seawater for nanometer-scale secondary ion mass spectrometry (nanoSIMS) were also taken [37].

At Stations 3, 5, 7, 9, 11, and 13 we examined the effects of nutrient amendments on CO_2_ fixation rates in surface waters (20 m). Incubations included: (N) 1 µM ammonium (as (NH_4_)_2_SO_4_) plus 1 µM nitrate (as NaNO_3_); (NP) a set of incubations with N additions as above and NaH_2_PO_4_ to a final phosphate concentration of 0.2 µM; (NFe) N additions as above in combination with Fe (elemental Fe dissolved in 10 M HCl) to a final Fe concentration of 2 nM; (PFe), phosphate and Fe additions at the same concentrations described above; (NPFe) nitrogen, phosphate and Fe in concentrations as described above; and (DW) 100 ml of water from 2500 m depth. Samples were processed as described above for the regular CO_2_ fixation experiments.

### Cell enumeration of *Prochlorococcus* and small photosynthetic eukaryotes

Cell numbers for *Prochlorococcus* and small photosynthetic eukaryotes (1–5 µm) were obtained by an Accuri C6 flow cytometer equipped with a blue laser (488 nm). Samples were run immediately upon collection of water samples from the CTD. Unstained cells were analyzed first by collecting signals for 5 min at a rate of 66 µl min^−1^ and by gating on forward scatter (FSC) and chl *a* fluorescence (FL3). Nano- and pico-eukaryotes were distinguished by size using FSC (Fig. S3). *Prochlorococcus* populations with dim chlorophyll fluorescence were tentatively identified by gating on green fluorescence (FL1) and chl *a* fluorescence (FL3) after staining with SYBR Green (Molecular Probes S7585) (Figs. S4–S8). Due to potential inaccuracies in counting using an Accuri C6 (BD Biosciences) flow cytometer in oligotrophic environments *Prochlorococcus* [39], cell numbers were cross-checked with direct cell counts and onboard contemporaneous DNA sequence reads [30]. Cell numbers in all treatments were calculated by the calibration with AccuCount Ultra Rainbow Fluorescent Particles (Spherotech).

### Single-cell sample imaging, nanoSIMS measurements, and calculations

*Prochlorococcus* cells were identified by catalyzed reporter deposition-fluorescence in situ hybridization (CARD-FISH) [40] with 15% formamide concentration during hybridization and probe PRO405 (ref. [41].). Positively hybridized *Prochlorococcus* cells and small photosynthetic eukaryotic cells (identified by chloroplast autofluorescence) were marked using a laser micro-dissection microscope (DM 6000 B, Leica Microsystems) and epifluorescence was imaged with a Axiocam 506 mono (Zeiss) or Axiocam MRm camera (Zeiss) for orientation during nanoSIMS analysis.

Isotopic compositions of marked cells were determined with a nanoSIMS 50 L ion microprobe (CAMECA, France) at two depths (surface, i.e., ~20 m, and chl *a* max) at Station 4, 8, and 12. Secondary ion images of ^12^C^−^, ^13^C^−^, ^19^F^−^, ^12^C^14^N^−^, ^12^C^15^N^−^, ^31^P^−^, and ^32^S^−^ were simultaneously recorded [37] (see Supplementary Information for instrument settings), and ^13^C/^12^C ratios and cell dimensions were calculated using Look@NanoSIMS [42]. Empirical biovolume-carbon relationships were used to calculate carbon content for each cell for small eukaryotes [43] and *Prochlorococcus* [44]. Carbon-based growth rates were estimated based on the incorporations of DI^13^C into biomass assuming exponential growth [37].

### DNA extraction, 16S rRNA metabarcoding, and *petB* gene mapping

Two liters of seawater were filtered onto polyvinylidene fluoride membrane filters (0.22 µm pore size; 47 mm diameter, Millipore), frozen immediately and stored at −80 °C. DNA was extracted using the Qiagen RNA/DNA Mini-Kit, after crushing the filter under liquid N_2_ with a sterile pipette tip, with an additional lysozyme step (200 µl of 5 mg ml^−1^ lysozyme solution for 10 min) prior to the addition of the kit lysis buffer. Partial 16S rRNA genes were amplified using previously described primers targeting the V4–V5 variable region of the 16S rRNA gene (515fb: GTGYCAGCMGCCGCGGTAA; 926 R: CCGYCAATTYMTTTRAGTTT) [45, 46]. Tag sequencing of DNA amplicons was carried out on an Illumina MiSeq instrument using 2 × 300 bp paired-end v3 chemistry at the Integrated Microbiome Resource, Dalhousie University [47]. The 16S rRNA gene amplicon sequence reads were processed using a QIIME1-based workflow (https://github.com/LangilleLab/microbiome_helper/wiki/16S-Bacteria-and-Archaea-Standard-Operating-Procedure) [48] available from the Microbiome Helper repository [47, 49]. Briefly, paired-end reads were merged using PEAR [50] and sequences <400 bp or with quality <30 over 90% of bases were discarded. Chimeras were removed using VSEARCH [51]. The programs SortMeRNA [52] and Sumaclust [53] were used for selecting operational taxonomic units (OTUs) (defined by 97% sequence similarity) identified as chloroplasts using Greengenes (16S rRNA) as a reference database [54]. Sequencing bleed-through was minimized by removing OTUs with a relative abundance of <0.1% [47]. All 618 chloroplast OTUs sequences obtained were also identified as chloroplasts when cross-checking against the SILVA SSU database (version 138) (ref. [55].).

Gene amplicon reads that mapped to chloroplasts in the Greengenes database were then annotated using the PhytoREF chloroplast database [56–58] with updated taxonomy in accordance to the integrated version in the PR2 version 4.12.0 database [57] as a reference. Reads were rarefied to 400 reads for all further analyses, and 7 of 71 original samples with <400 reads mapped to chloroplasts were discarded. Phylogenetic analysis was performed with the ARB software package [59]. Selected sequences from the PhytoREF chloroplast database [56] were used as reference sequences [57, 58]. We calculated a tree with the top 50 chloroplast 16S rRNA OTUs from this study with selected reference sequences (further details in the Supplementary Information).

Shotgun metagenomes were generated for eight samples from the core of the SPG using extracted DNA as described above. Library preparation and sequencing were performed at the Max-Planck Genome Center Cologne, Germany (https://mpgc.mpipz.mpg.de/home/). To assess phytoplankton community composition in shotgun metagenomes, 16S and 18S rRNA reads were mapped onto reference databases using phyloFlash [60]. The PR2 version 4.12.0 database [57] was used for 18S rRNA based analysis. For 16S rRNA a custom database was created comprising all bacterial and archaeal entries of the SILVA SSU database (version 138) [55] together with the PhytoREF chloroplast 16S rRNA database [56], with updated taxonomy in accordance to the integrated version in the PR2 version 4.12.0 database [57]. We removed non-chloroplast sequences after checking the 16S rRNA metagenomic data, as well as sequences from the class of Embryophyceae (land plants), which were assumed to be contaminants.

In order to assess the distribution of *Prochlorococcus* ecotypes, we mapped the metagenomic reads from the metagenomes of the gyre region to a custom database of the petB gene, a high-resolution taxonomic marker for *Prochlorococcus* ecotypes [61]. We were also afforded access to an additional unpublished set of metagenomes that were generated from in situ pump samples collected contemporaneously during the SO245 expedition [62] (see Supplementary Information for more detail on collection and processing). The custom-made petB gene database [61] consisted of the *Prochlorococcus* subset. Trimmed reads were mapped against the database using bbmap at ≥99% identity. Calculated Reads per Kilobase per 10^6^ Mapped Reads (RPKM) of all entries were summed within each ecologically significant taxonomic unit (ESTU).

### Data Visualization, community analysis, and data deposition

Data were visualized using Ocean Data View [63], the R [64] package “ggplot2” [65], and Look@NanoSIMS [42]. Differences in the photosynthetic community structure between sample sites were analyzed and visualized by nonmetric multidimensional scaling (NMDS) plots with Bray–Curtis dissimilarity. A redundancy analysis (RDA) of the environmental parameter (CO_2_ fixation rate, PAR, DIN, chl *a* concentrations and salinity) was performed using only samples for which all metadata were available using the R package “phyloseq” [66]. A Hellinger transformation for the rarefied chloroplast 16S rRNA gene amplicon dataset was performed prior to the analysis. The environmental parameters were normalized with z-scoring using the R package “vegan” 2.5 (ref. [67]).

16S rRNA amplicon metabarcodes, metagenome 16S rRNA and 18S rRNA, and metagenome *petB* gene read sequences are deposited in NCBI Sequence Read Archive under Bioproject: PRJNA670604 (https://www.ncbi.nlm.nih.gov/bioproject/). The chloroplast metabarcode accession codes for this project are: MW152420-MW153037. Physical oceanographic data (10.1594/PANGAEA.890394) (ref. [31].), light data (10.1594/PANGAEA.911558) (ref. [36].) and nutrient data (10.1594/PANGAEA.899228) (ref. [32]) were deposited in the Pangaea Data Publisher. Flow Cytometry Data can be accessed at the Flow Repository Databank (https://flowrepository.org/id/RvFrru2M2yxF4dtwRMw1hAgFPMmQdqgd9Ki3Kamo4FWdAhzjjCkAhAR6sapm3c6Q).

Supplementary Files containing station list sampling depths and CO_2_ fixation data (Supplementary File 2), single-cell rate calculations (Supplementary File 3) and results of the statistical evaluation of the nutrient addition experiment (Supplementary File 4) are publicly accessible in xlsx or csv formats from the MPG Data Repository (EDMOND; 10.17617/3.6q). The extracted alignment for the the 16S rRNA chloroplast tree (Supplementary File 5) is also available as a text file in this collection. Table S1 provides and an overview of data sets and repository sites.

## Results

### Physical and chemical properties

We identified two principal oceanographic environments based on chl *a*, salinity and DIN distributions during the UltraPac expedition: (a) the oligotrophic waters of the SPG (Stations 2 through 10) and (b) the mesotrophic waters of the SWP (Stations 11–15) located south and west of the South Pacific Subtropical Front (SPSF) (Figs. 1, S9). (Station 1 in the Eastern South Pacific (ESP), although oligotrophic, lies outside the core of the SPG with respect to surface chl *a* concentrations.) The observed salinities exceeding 35 PSU at Stations 1 through 10 extending down to depths of 250 meters in the center of the gyre (Fig. 1c) define the core SPG waters [68] which exhibit strongly depleted DIN concentrations (<0.1 µmol l^−1^, Fig. 1e). DOC concentrations were >80 µmol l^−1^ throughout the core SPG with concentrations of up to 107 µmol l^−1^ in near surface waters (Fig. 1g). Fluorometric determination of chl *a* concentrations on discrete water samples were consistent with the CTD-derived fluorescence distributions [30], and confirmed the extremely low concentrations of surface chl *a* concentrations detected in the center of the SPG via satellite (Fig. 1a, b). Surface chl *a* concentrations at Stations 2 through 10 were below 0.1 µg l^−1^, which correspond to the criteria for an oligotrophic environment [8] (Fig. 1a, b). High photosynthetically active radiation (PAR) intensities of >1000 µmol photons m^−2^ s^−1^ at the surface to 100 µmol photons m^−2^ s^−1^ at depths of 100 m characterized the upper euphotic zone of the oligotrophic SPG core (Figs. 1d, S2a). The chl *a* max in the gyre lay just below the 10 µmol photons m^−2^ s^−1^ isopleth measured at water depths below 150 m (Fig. 1d). The chl *a* max (as defined by the 0.5 µg chl *a* l^−1^ isolines in Fig. 1b) deepened towards the center of the gyre with a maximum depth of about 200 m at Station 7. Within the easternmost stations (Stations 1 through 4), and at the southwestern periphery of the SPG (Station 10) the nitricline (1 µmol DIN l^−1^), the PAR 10 µmol photons m^−2^ s^−1^ isopleth, and chl *a* max shallowed to depths of 100 m (Fig. 1b, d, e).

The drop in surface water salinity from 35 to 34.5 PSU between Stations 10 (33.5°S) and 11 (36°S) marked the transition to the mesotrophic waters present south of the SPSF (Fig. 1c). In the mesotrophic waters of the SWP south of the SPSF (Stations 11 to 15) along the southwestern periphery of the SPG, the chl *a* max continued to broaden and shoal towards the surface (ca 25 to 125 m) (Fig. 1b). PAR intensities of 10 µmol photons m^−2^ s^−1^ penetrated only to depths of 75 m (Fig. 1d). Likewise, the nitricline (DIN <1 µmol l^−1^) was located at depths less than 50 m (Fig. 1e). DOC concentrations in the SWP were 10 to 20 µmol l^−1^ lower than in the SPG (Fig. 1g).

### Rates of CO_2_ fixation

**Fig. 2:**
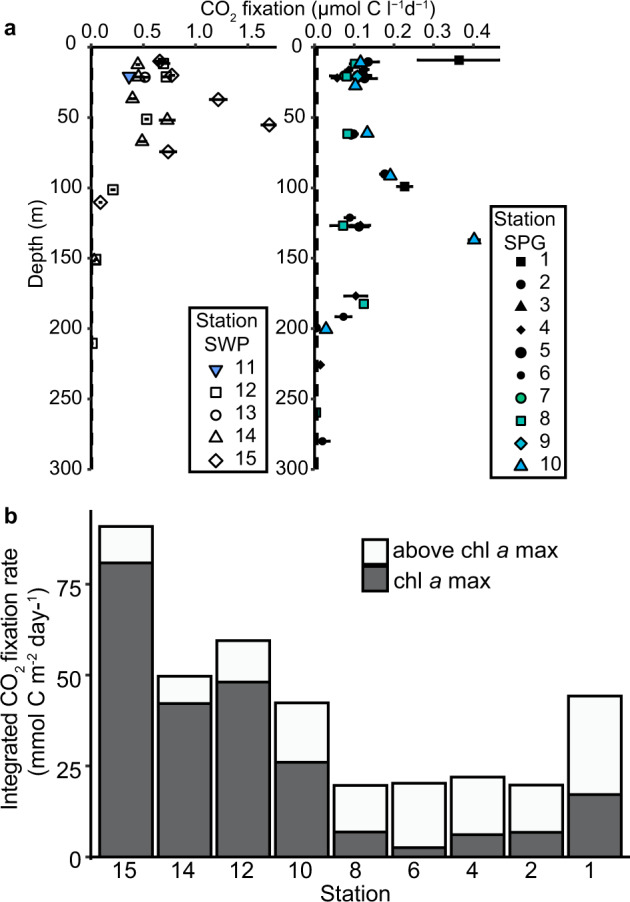
Vertical profile of CO_2_ fixation at all sample stations in the Southwest Pacific (SWP) and South Pacific Gyre (SPG) and depth-integrated CO_2_ fixation rates. **a** Vertical CO_2_ fixation rates. Horizontal bars represent the standard deviation of the triplicate measurements, in most cases the one standard deviation error bars are smaller than the symbols. The minimum quantifiable limit (average 0.007 µmol C l^−1^ d^–1^ for all samples) is indicated by the vertical dashed line along the *y*-axis. **b** Depth-integrated CO_2_ fixation rate from surface to below the chl *a* max. White indicates the CO_2_ fixation above the chl *a* max and gray the CO_2_ fixation within the chl *a* max. Station 1 rates were integrated only over two depths rather than six as in the other stations).

**Fig. 3:**
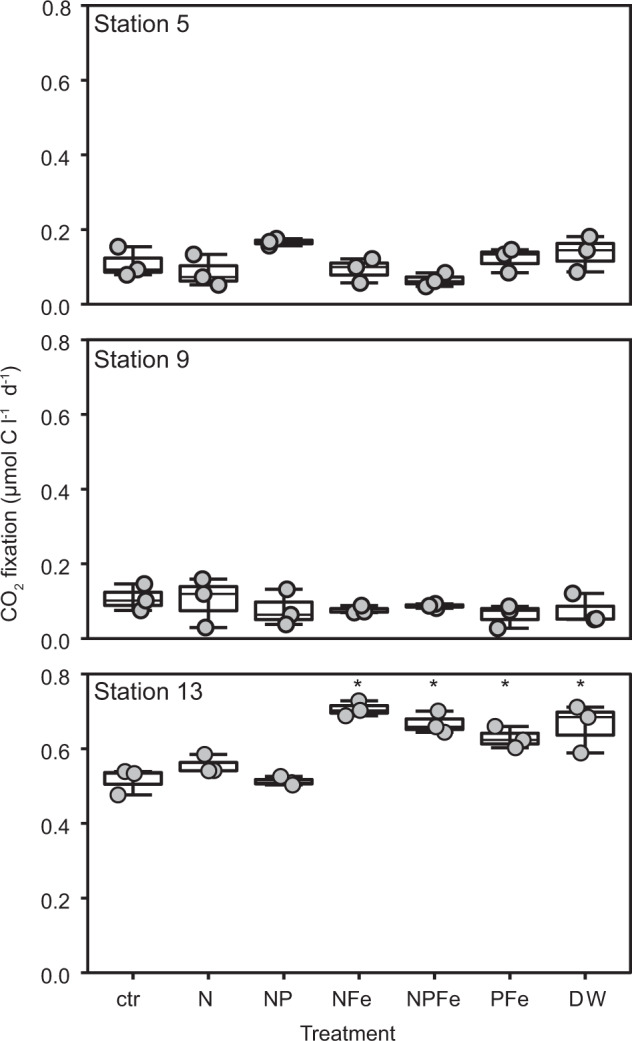
Surface water (20 m) nutrient manipulation experiments. CO_2_ fixation rates from nutrient addition experiments on samples amended with single or combinations of nutrients, where: ctr (control without any addition), N (addition of NH_4_^+^ and NO_3_^−^), P (addition of PO_4_^3−^), dFe (dissolved Fe), and deep water (DW; obtained from 2500 m water depth). Treatments were compared using one-way ANOVA and a Tukey multiple pairwise comparison test; means that are significantly different from the control are labeled with an asterisk (*p* < *0.05*).

Rates of CO_2_-fixation based on DI^13^C uptake in the oligotrophic SPG euphotic zone differed in both magnitude and depth distribution as compared to the adjacent mesotrophic SWP (Figs. 1f, 2a). Volumetric CO_2_ fixation rates in the mesotrophic SWP covaried with chl *a* distributions and were higher (0.2–1.71 µmol C l^−1^ d^−1^) than in the core of the oligotrophic SPG (~0.1 µmol C l^−1^ d^−1^) (Figs. 1b, f, 2a). Only at the edges of the gyre (Stations 1 and 10) did the rates of CO_2_ fixation exceed 0.2 µmol C l^−1^ d^−1^. In contrast to the mesotrophic SWP, rates of CO_2_ fixation in the core of the oligotrophic SPG (Stations 4 through 8) were evenly distributed across all depths from the surface to the chl *a* max (Figs. 1f, 2a) and independent of light levels (Figs. 1d, f, S10). Depth-integrated rates of CO_2_ fixation in the SWP (50 to 95 mmol C m^−2^ d^−1^) exceeded by two to fivefold rates in the core of the gyre (20 mmol C m^−2^ d^−1^) (Fig. 2b). On the eastern (Station 2) and southwestern peripheries (Station 10) of the SPG, areal rates of CO_2_ fixation were slightly enhanced, consistent with the shallowing nutricline (Fig. 2b). Various nutrient additions to surface water incubations (20 m water depth) from the oligotrophic SPG failed to stimulate ^13^CO_2_ uptake relative to the controls over a 24 h period (Figs. 3, S11). A significant increase upon nutrient additions was observed only in the mesotrophic samples from Station 13, and only in the Fe- or deep water-amended experiments (Figs. 3, S11).

### Distributions of *Prochlorococcus* and small photosynthetic eukaryotes

**Fig. 4:**
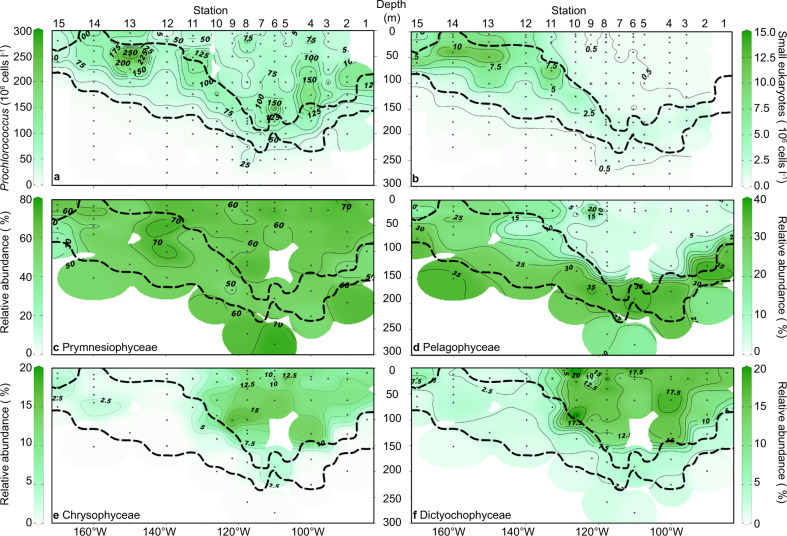
Distribution of dominant photosynthetic organisms along the SO245 UltraPac transect. Numerical abundances of (**a**) *Prochlorococcus* and (**b**) small photosynthetic eukaryotes. Relative abundances of chloroplast 16S rRNA gene OTUs (% of total chloroplast sequences, rarefied at 400 reads per sample) are shown for (**c**) Prymnesiophyceae, (**d**) Pelagophyceae, (**e**) Chrysophyceae and Synurophyceae, and (**f**) Dictyochophyceae. Dots and dashed lines as in Fig. 1.

*Prochlorococcus* was the most abundant photosynthetic organism and exhibited a 10-fold greater abundance than small photosynthetic eukaryotes (Figs. 4a, b, S3). In highly oligotrophic environments, accurate determination of low chlorophyll content picocyanobacteria such as *Prochlorococcus* is difficult and subject to potential underestimation [39]. The *Prochlorococcus* abundances determined with flow cytometry, however, were consistent with 16S rRNA contemperaneous read abundances and FISH count results [30] (and Fig. S12). Abundances of *Synechococcu*s (3.8 × 10^3^ to 2 × 10^6^ cells l^−1^) rarely exceeded abundances of the small eukaryotes and were often orders of magnitude lower than abundances of *Prochlorococcus* (Figs. S3–S6) consistent with other measurements [30]. Peak densities of *Prochlorococcus* in the oligotrophic SPG were observed above the chl *a* max band (up to 2 × 10^8^ cells l^−1^, Fig. 4a), whereas the surface abundance of *Prochlorococcus* was never greater than 7.3 × 10^7^ cells l^−1^ in the oligotrophic SPG (Fig. 4a). *Prochlorococcus* was also present throughout the euphotic zone of the SWP, with peak abundances of 2.5 × 10^8^ cells l^−1^ in the chl *a* max at Station 13 (Fig. 4a). Relative abundances of *Prochlorococcus* 16S rRNA reads (Fig. S13a) were also qualitatively consistent with overall cell abundances, with greater relative abundances more closely associated to the chl *a* maximum, and depleted relative abundances in the surface waters of the core of the SPG. We were able to retrieve 189 of the original 190 entries in the petB database [61], of which 6 ESTUs dominated (RPKM values >3; Fig. S13b). ETSUs associated with *Prochlorococcus* ecotype HLIA dominated the surface waters along the outer rims of the SPG, even down to water depths of 170 m (Station 4). They were noticeably absent from core ultraoligotrophic SPG waters (Stations 6–8). The high-light ecotypes HLIIA and HLIIB, on the other hand, were equally distributed throughout all depths >200 m. Low-light ecotypes, LLIA, C, and D were closely associated with the deep chl *a* max.

Small photosynthetic eukaryotes (1–5 µm; Figs. 4b, S14) were more closely associated with the chl *a* max (1.62 × 10^6^ to 4.68 × 10^6^ cells l^−1^, Fig. 4b) of the oligotrophic SPG. Absolute abundances of photosynthetic eukaryotes were elevated in the mesotrophic SWP waters (up to 1.3 × 10^7^ cells l^−1^) in contrast to the low abundances of small eukaryotes in the surface of the oligotrophic SPG (<9.1 × 10^5^ cells l^−1^, Fig. 4b). The analysis of the chloroplast 16S rRNA metabarcoding revealed 618 distinct OTUs, about 10% of total reads. Results from the 16S rRNA chloroplast metabarcoding were in good agreement with the 16S rRNA gene sequences retrieved from metagenomes for eight water samples in the upper ~60 m within the SPG (Fig. S15). The metagenome approach, which is more sensitive and less biased against certain groups [69], yielded greater diversity and relative abundances of Bacillariophyceae and Prasino-Clade-9 at the expense of Prymnesiophyceae and Chloropicophyceae (Fig. S15). Overall, however, both approaches yielded similar relative abundance patterns among the Dictyochophyceae, Chrysophyceae and Pelagophyceae (Fig. S15). Overall eukaryotic diversity in the upper ~60 m within the SPG, assessed using 18S rRNA gene sequences retrieved from the eight metagenomes, revealed a high diversity of eukaryotes, including photo-and heterotrophic organisms, inhabiting the surface waters of the SPG (Fig. S16). The photosynthetic community showed a greater abundance of Chlorophyceae and general larger organisms such as Euglenozoa and the metabolic diverse group Dinophyceae (Fig. S15).

**Fig. 5:**
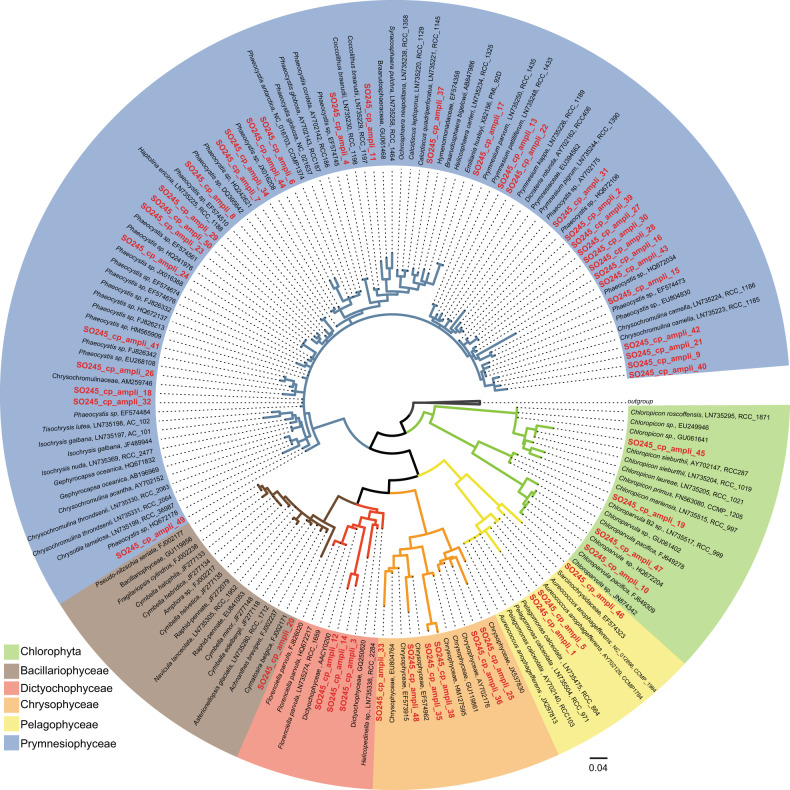
Phylogenetic tree of chloroplast 16S rRNA gene sequences. The tree represents the top 50 OTUs from the SO245 cruise (in bold and red) and selected reference sequences drawn from the PhytoREF database [56], the first number represents the PhytoREF accession number and the second number the original ID. The tree was constructed based on maximum likelihood (RAxML7) with 66 long reference sequences with 1265 valid positions (sequences were at least 1300 nucleotides long). Other partial reference sequences and the 53 partial 16S rRNA chloroplast gene sequences from this study were added to the tree constructed with the longer reference sequences.

Prymnesiophyceae dominated the relative chloroplast gene amplicon relative abundance (up to 70%), and were the only OTUs ubiquitously present throughout the entire transect (Fig. 4c). More than one-half of the OTUs (393) were affiliated with the Prymnesiophyceae. Most of the Prymnesiophyceae OTUs were closely related to sequences assigned to the genus *Phaeocystis* (up to 65% of the Prymnesiophyceae) that was broadly distributed throughout the South Pacific (Figs. 4c, S14d). The family Chrysochromulinaceae was distributed throughout the whole transect, but with higher relative abundances in the core of the SPG (Fig. S14f). Diatom OTUs (Bacillariophyceae) (Fig. S14b) were mostly found in the chl *a* max and in the mesotrophic waters of the SWP. The core SPG waters above the chl *a* max were additionally characterized by greater relative abundances of Dictyochophyceae (up to 17.5% Fig. 4f), Chrysophyceae (up to 15% relative OTU abundance, Fig. 4e). Near relatives to the Chrysophyceae, for which there are only a few described species [70, 71], were found at the class level (Fig. 5). Abundant OTUs affiliated to the class of Dictyochophyceae were most similar to the genus *Helicopedinella* and the species *Florenciella parvula* (Fig. 5).

**Fig. 6:**
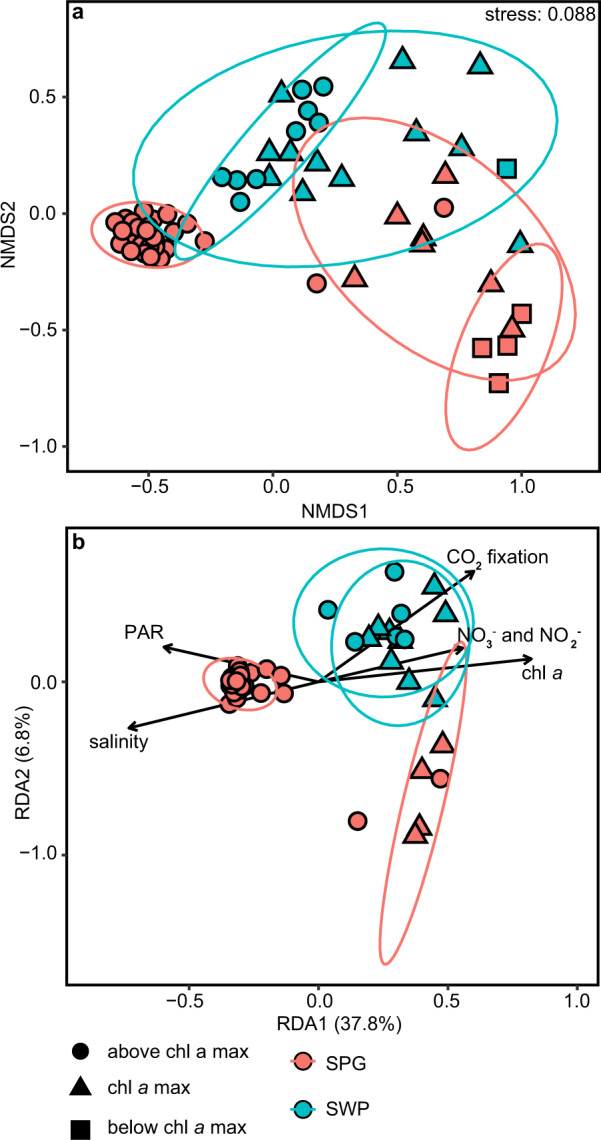
Community composition analysis based on the abundances of chloroplast 16S rRNA genes. **a** Nonmetric multidimensional scaling (NMDS) plot showing Bray–Curtis dissimilarity in community composition. **b** Redundancy analysis (RDA) of the community composition and environmental variables measured at the sampling Station. Each shape represents an individual sample and the communities are coded according to the legend.

Within the chl *a* max, Prymnesiophyceae and Pelagophyceae dominated relative read abundances in both the mesotrophic SWP and the oligotrophic SPG (Fig. 4c, d). The class Pelagophyceae was dominated by sequences, which were closely associated with *Pelagomonas* spp. (Figs. 5, S14h). The most abundant OTU (SO245_cp_1) in this dataset was most similar to *Pelagomonas calceolata* (Fig. 5). Chlorophyta comprised 10 to 20% of the relative OTU abundance within the chl *a* max of the SPG (Fig. S14a), and was most closely associated with the genera *Chloropicon* and *Chloropavula* within the class Chloropicophyceae (Figs. 5, S14c). The nonmetric multidimensional scaling (NMDS) analysis of the chloroplast 16S rRNA gene amplicon community composition shows distinct clustering of OTUs associated with the SPG core waters above the chl *a* max (Fig. 6a) that is strongly associated with high PAR and low nutrient concentration as shown by further redundancy analysis (RDA; Fig. 6b). Outliers from this trend include one sample at Station 2 very close to the chl *a* max, and one sample from Station 9 at surface waters.

### Single-cell CO_2_ fixation by *Prochlorococcus* and small photosynthetic eukaryotes

**Fig. 7:**
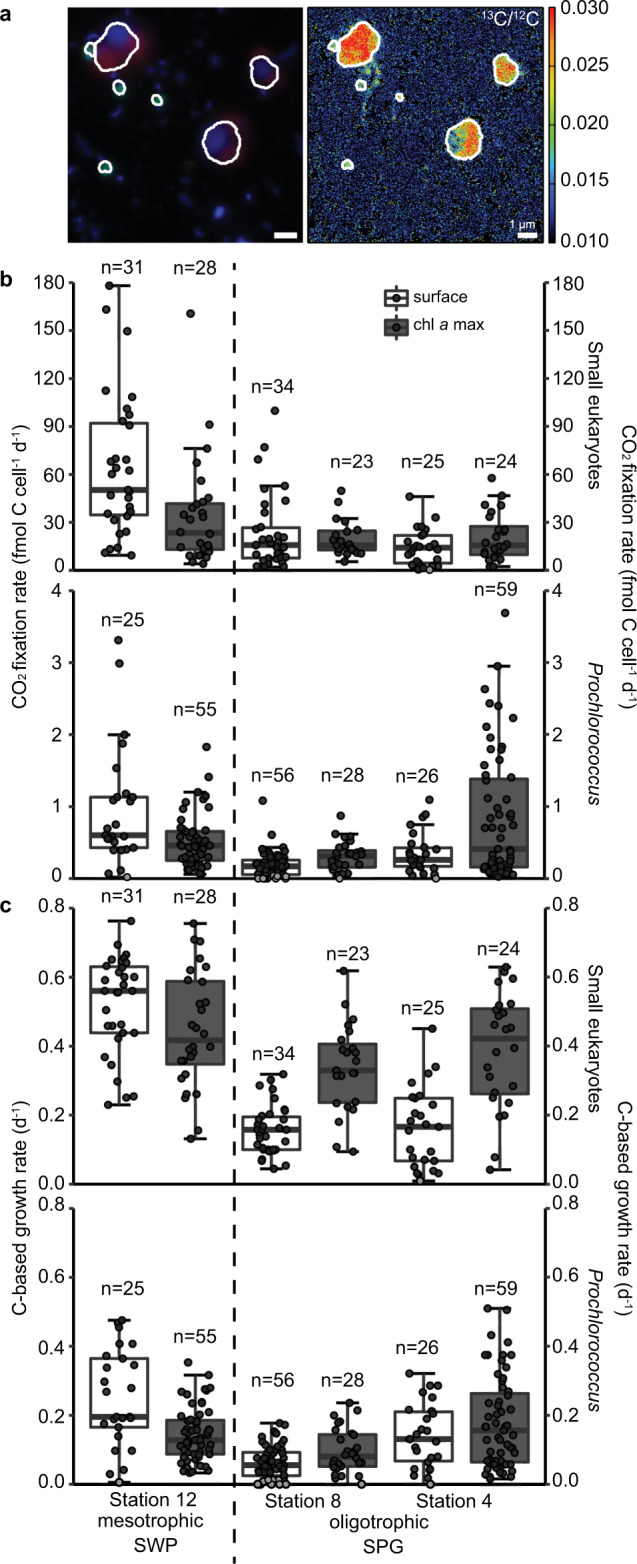
Single-cell imaging and activity in the surface waters (20 meter water depth) and in the chl *a* max at two sites in the oligotrophic SPG (Stations 4 and 8) and one site in the mesotrophic SWP. **a** Fluorescence in situ hybridization image (green: CARD-FISH probe PRO405, red: autofluorescence, blue: DAPI signal) of *Prochlorococcus* and small photosynthetic eukaryotes from the chl *a* max at Station 12 and ^13^C/^12^C ratio image from nanoSIMS analysis; (**b**) single-cell CO_2_ uptake, and (**c**) C-based growth rate of small photosynthetic eukaryotes and *Prochlorococcus*. Black dots indicate measurements above the detection limits, while the gray dots were below the detection limit. For each boxplot: dark horizontal line indicates the median, the box boundaries span the 1st (25th percentile) to the 3rd quartile (75th percentile), and the whiskers encompass data points within 1.5× interquartile range of the selected measurements. Results of statistical evaluation can be found in the Supplementary File 4.

Single-cell ^13^C incorporation of ^13^CO_2_ was used to assess the CO_2_ fixation rates of individual photosynthetic eukaryotes and *Prochlorococcus* cells at two stations within the oligotrophic SPG (Stations 4 and 8) and one in the mesotrophic SWP (Station 12). Small photosynthetic eukaryotes and *Prochlorococcus* cells were enriched in ^13^C, demonstrating active ^13^CO_2_ fixation during the 24 h incubations in the surface waters and in the chl *a* max at all three stations (Fig. 7a–c). In the gyre, single-cell rates of ^13^CO_2_ fixation by the small eukaryote fraction centered around 15 fmol C cell^−1^ d^−1^ at all depths (Fig. 7b, Table S2; Supplementary File 3), whereas, the single-cell CO_2_ fixation rates for the eukaryotes were enhanced in the waters of the SWP. Median values in the SWP were 50 fmol C cell^−1^ d^−1^ and 23 fmol C cell^−1^ d^−1^, for surface and chl *a* max, respectively. *Prochlorococcus* single-cell activity exhibited enhanced cell-specific rates of CO_2_ uptake at the edges of the SPG, with highest rates in the chl *a* max of Station 4 of 0.52 fmol C cell^−1^ d^−1^ and 0.6 fmol C cell^−1^ d^−1^ in the SWP, where DIN fluxes are expectedly greater. In contrast median values of <0.3 fmol C cell^−1^ d^−1^ were observed in the core oligotrophic gyre waters at Station 8 (Fig. 7b). Moreover, 20% of *Prochlorococcus* cells within Station 8 surface waters showed no detectable activity. Within the Station 4 chl *a* max, the median cell-specific CO_2_ fixation rates for *Prochlorococcus* were low, but the range of individual cellular rates showed a large degree of skew towards high rates of C uptake (Fig. 7b, Table S2; Supplementary File 3). C-based growth rates for *Prochlorococcus* likewise exhibited the greatest range of growth rates in the chl *a* max of Station 4 (median: 0.16 d^−1^) where the nutricline shoals towards the surface. Conversely, C-based growth rates for *Prochlorococcus* were low in both surface and chl *a* max waters in the oligotrophic SPG (0.06 to 0.8 d^−1^) (Fig. 7c, Table S2; Supplementary File 3). C-based growth rates for the small photosynthetic eukaryotes in the oligotrophic SPG Stations 4 and 8 exhibited marked differences between the surface waters (0.16 to 0.17 d^−1^) and the deep chl *a* max, where C-based growth rates (0.33 to 0.39 d^−1^) for photosynthetic eukaryotes approached those of the mesotrophic SWP (0.42–0.56 d^−1^).

Overall volumetric rates of *Prochlorococcus* CO_2 _fixation in the center oligotrophic SPG (Station 8) were very low (0.006 to 0.02 µmol C l^−1^ d^−1^), and lower than volumetric rates for small photosynthetic eukaryotes, which ranged from 0.01 µmol C l^−1^ d^−1^ at the surface to 0.03 µmol C l^−1^ d^−1^ in the chl *a* max (Fig. S17, Table S2; Supplementary File 3). The contribution of *Prochlorococcus* to primary production was indeed only greater at Station 4 with 0.019 ± 0.021 µmol C l^−1^ d^−1^ and 0.05 ± 0.021 µmol C l^−1^ d^−1^ at the surface and chl *a* max, respectively, (32 and 44.4% of total productivity). In the mesophilic SWP, volumetric rates of CO_2_-fixation by small photosynthetic eukaryotes (0.05 to 0.28 µmol C l^−1^ d^−1^) were also substantially greater than that of *Prochlorococcus* (0.02 to 0.04 µmol C l^−1^ d^−1^).

## Discussion

The SPG is often referred to as being ultraoligotrophic or an “oceanic desert” [26] and studies on primary production in the SPG indicate that rates of biological CO_2_ fixation are indeed low (~0.08–0.17 µmol C l^−1^ d^−1^) [27–29, 72]. More importantly, our data show that CO_2_ fixation is pervasive and occurs at similar rates throughout all water depths within the euphotic zone, i.e., not just in the chl *a* maximum. This contrasts with other ocean gyres, where CO_2_ fixation activity distributions often peak just above the deep chl *a* max [15, 73, 74]. Volumetric rates may be low in the SPG, but the unparalleled depth of the euphotic zone in the SPG leads to depth-integrated CO_2_ fixation rates that match those of other ocean gyre ecosystems (~20 mmol C m^−2^ d^−1^; Fig. 2). Moreover, 65 to 88% of the areal CO_2_ fixation occurs in the ultraoligotrophic, low chl *a*, nutrient-deplete waters above the chl *a* maximum. In the following discussion, we address how niche partitioning and CO_2_ uptake activity among both the small photosynthetic eukaryote and *Prochlorococcus* communities appear to compensate for changing light and nutrient fluxes, and thus, maintain the low but pervasive rates of CO_2_ fixation rates across all depths of the SPG euphotic zone.

NMDS and redundancy analysis (RDA) of community composition reveal that the SPG euphotic zone encompasses two distinct niches (Fig. 6). These consist of (1) an upper well-mixed, high-light intensity, high salinity, and nutrient-deplete layer that drives three quarters of the depth-integrated CO_2_ fixation and (2) the thinner, density-stratified, low-light, deep chl *a* max layer that sits on the nutricline. The upper mixed layer of the euphotic zone is distinguished by the presence of members of the Chrysophyceae, Dictyochophyceae (Fig. 4e, f) and the genus *Phaeocystis* (Fig. S14d), consistent with earlier studies [70, 75–78], and low relative abundances of *Prochlorococcus* ecotype HLIIA&B. The relative abundances of these groups also correspond to the broad zone dissolved inorganic C depletion identified the zone of significantly contributing to the net export of biologically fixed CO_2_ (as particles or DOC) out of the euphotic zone of the SPG (i.e., so-called net community or export production) [79].

The density structure and physical mixing characteristics of the upper mixed-layer abet the broad distribution of CO_2_ fixation activity and abundance. The upper 175 m of the SPG becomes well mixed to depths below 175 m in the winter and only shallows in late summer (March–April) [80, 81]. Furthermore, mixed-layer stratification remains weak due to compensating temperature and salinity gradients plus salt-fingering that leads to enhanced mixing [82]. Such mixed-layer properties sustain a large, homogenous niche with respect to the bulk CO_2_ fixation activity and distribution of photosynthetic plankton.

The single-cell ^13^CO_2_ uptake experiments and nutrient amendment experiments provide insight into how this community may function within the SPG mixed layer. The nutrient addition experiments suggest that the CO_2_-fixing community inhabiting the surface waters (20 m water depth) of the oligotrophic SPG is strictly adapted to extremely low fixed-nitrogen and iron fluxes (Figs. 3, S11) of the mixed layer. Despite DIN and presumably Fe limitation (phosphate is in excess), we observed no significant response to any combination of inorganic nutrient addition in short-term (24 h) experiments designed to test for constitutive nutrient uptake. This contrasts to the immediate and positive response to amendments containing dissolved iron in the more productive SWP waters (Figs. 3, S11) that suggests that the SWP community is poised to take up inorganic sources of Fe (and perhaps N). DIN and Fe fluxes to the SPG community, in contrast, are vanishingly small [83, 84]. Although positive responses to DIN additions have been recorded in longer (48–72 h) incubations [85, 86], minimal responses to nutrient additions, as observed in our experiments, have also been noted in short-term incubations at oligotrophic sites in the Atlantic [87]. This and other studies argue that short-term nutrient addition experiments reveal that photosynthetic plankton in highly oligotrophic environments grow close to their maximum potential growth rates and thus are unable to respond immediately to nutrient inputs [87–89].

Our single-cell ^13^CO_2_ uptake experiments show that single-cell CO_2_-based growth and fixation rates for *Prochlorococcus* and small photosynthetic eukaryotes are indeed extremely low in the surface waters at the core SPG station 8 (Fig. 7). One fifth of the analyzed *Prochlorococcus* cells did not actively take up labeled ^13^CO_2_. Similar observations of depressed CO_2_ uptake activity have also been observed for *Prochlorococcus* in the North Pacific Gyre and California Current system (where 13 and 53% of cells were inactive) [90]. *Prochlorococcus* may also partially fulfill their C and N demands through organotrophic uptake [88, 91, 92], and we note that previous short-term (12 h) leucine addition experiments in the SPG stimulated bulk CO_2_ fixation [27]. Low per cell CO_2_ fixation rates, low C-based growth rates, and lack of immediate, constitutive response to inorganic nutrient additions in the bulk ^13^C experiments thus point to a primary producer community attuned to the recycling of organically bound nutrients within the mixed-layer zone of the core SPG.

In contrast to the DIN-depleted waters of the core SPG, single-cell ^13^CO_2_ uptake experiments show that *Prochlorococcus* exhibits enhanced CO_2_-uptake and growth within the eastern boundary chl *a* max of the SPG at Station 4 (Fig. 7). These enhanced rates correspond to higher relative abundances of *Prochlorococcus* ESTUs associated with the HLIA *Prochlorococcus* ecotype along both the eastern and western boundaries of the SPG, where the nutricline shoals towards the surface (Fig. S13c). Moreover, the most abundant Low-Light ecotype ESTUs exhibit high relative abundances in the SPG where the chl *a* max straddles the nutricline (Fig. S13b). Light appears to not play a predominate role, but rather the proximity of greater nutrient fluxes associated with the nutricline appears to dictate the distribution and CO_2_ fixation activity of *Prochlorococcus* in the SPG and SWP. Contributions by the cyanobacterial *Prochlorococcus* to the total CO_2_ fixation also indicate this niche preference (Fig. S17). *Prochlorococcus* abundances, activity and growth are more closely associated with the edges of the gyre where nutrient fluxes are enhanced, consistent with reports of decreasing importance of *Prochlorococcus* moving from the upwelling-influenced waters off northern Chile and into the oligotrophic SPG [75].

C-based growth rates are similar for small photosynthetic eukaryotes in the chl *a* max of both the SPG and SWP (Fig. 7c, Table S2; Supplementary File 3). They also exhibit growth rates twice as high as that in surface waters (Fig. 7c), yet per cell CO_2_ uptake rates are similar in the deep chl *a* max and surface waters (Fig. 7b). This discrepancy between growth rate and cell-specific C uptake can be attributed to the distinctly smaller cell biovolume, and thus C contents [43, 44] of the measured cells growing in the chl *a* max (Figs. S18–S19). Smaller cell biovolumes of photosynthetic eukaryotes in the chl *a* max also correspond to the high abundances of chloroplast bearing Pelagophyceae (Fig. 4d), and in particular *Pelagomonas* spp. chloroplast 16 S rRNA read distributions (Fig. S14h). *Pelagomonas calceolata*, whose mean size is about 2–3 µm, is a low-light-adapted species commonly found in the deep chl *a* max [93–95]. Our 16S rRNA sequencing data coincides with earlier18S rRNA gene distributions of phytoplankton [70, 75, 76], and both *Phaeocystis* and *P. calceolata* have been isolated from the SPG [96]. Overall, the chl *a* max of both the SPG and SWP share an overlap in the activities and distributions of both *Prochlorococcus* ecotypes and small photosynthetic eukaryotes, and whose distributions are dictated by their proximity to the nutricline.

The oligotrophic SPG thus comprises two distinct eukaryotic phytoplankton communities. In the deep chl *a* max, this consists of a fast-growing, but small sized community most likely dominated by the species closely related to the Pelagophyceae, *Pelagomonas* spp., and the classes of Prymnesiophyceae and Chlorophyceae with the genera *Chloropicon* and *Chloroparvula*. Within the well-lit but DIN-deplete mixed layer a slower growing population of photosynthetic eukaryotes dominated by populations of Dictyochophyceae and Chrysophyceae are present. The single-cell ^13^C analysis shows that larger mean biovolumes among small photosynthetic eukaryotes in the SPG mixed-layer compensate for their low ^13^CO_2_ uptake rates (Figs. S18–S19). This leads to substantial contributions by the small photosynthetic eukaryote community to the areal rates in the clear, low chl *a*, nutrient-deplete mixed-layer biological CO_2_-fixation (Fig. S17a; Table S2; Supplementary File 3). Thus, our detailed cross-basin transect through the SPG highlights the potential of small photosynthetic eukaryotes as important contributors to CO_2_-fixation in the Ocean’s most oligotrophic surface waters.

Our ability to predict ocean CO_2_ sequestration from the atmosphere depends on the accuracy of ocean biogeochemical models to predict, among other things, CO_2_ fixation into biomass and the export of this biomass into deeper layers of the ocean. These models, in turn, depend on accurate parameterization of satellite global color imagery, i.e., surface chl *a* concentrations, as a proxy for ecosystem structure and productivity [6, 11]. Recent niche modeling suggests that warming will increase phytobiomass in oligotrophic regions in the form of a shift towards slightly larger biovolumes among photosynthetic eukaryotes (e.g., increase in nano- and microphytobiomass) [6]. Our single-cell CO_2_ uptake data is consistent with this hypothesis. The highly abundant picocyanobacteria *Prochlorococcus* appears to be more abundant and active with regards to CO_2_ fixation along the oligotrophic rims of the SPG, for instance near frontal structures along the northern edge where the nutricline begins to shoal. In contrast, there is a shift towards an enhanced role for slow-growing, but larger photosynthetic eukaryotes in the ultraoligotrophic core of the SPG. CO_2_ fixation and associated increased rates of recycling of DON by photosynthetic eukaryotes in these DIN-deplete waters may enhance the DOC accumulation observed in the euphotic zone of the SPG [35] and eventual C export [13]. Thus, the niche partitioning among CO_2_ fixing phytoplankton as described in our study in the SPG can better inform models describing carbon flow through expanding ocean gyre ecosystems.

## Supplementary information

Supplemental File 1: Supplementary Information

Supplemental File 2: Station List and Rates Data

Supplemental File 3: Single Cell Rates Data

Supplemental File 4: Statistics to Figure 4

Supplemental File 5: Figure 5 Alignment Txt File

## References

[1] Irwin AJ, Oliver MJ (2009). Are ocean deserts getting larger?. Geophys Res Lett.

[2] McClain CR, Signorini SR, Christian JR (2004). Subtropical gyre variability observed by ocean-color satellites. Deep Sea Res Part II Topical Stud Oceanogr.

[3] Signorini SR, Franz BA, McClain CR (2015). Chlorophyll variability in the oligotrophic gyres: Mechanisms, seasonality and trends. Front Mar Sci.

[4] Polovina JJ, Howell EA, Abecassis M (2008). Ocean’s least productive waters are expanding. Geophys Res Lett.

[5] Sharma P, Marinov I, Cabre A, Kostadinov T, Singh A (2019). Increasing biomass in the warm oceans: unexpected new insights from SeaWIFS. Geophys Res Lett.

[6] Flombaum P, Wang W-L, Primeau FW, Martiny AC (2020). Global picophytoplankton niche partitioning predicts overall positive response to ocean warming. Nat Geosci.

[7] Carr M-E, Friedrichs MAM, Schmeltz M, Noguchi Aita M, Antoine D, Arrigo KR (2006). A comparison of global estimates of marine primary production from ocean color. Deep Sea Res Part II Topical Stud Oceanogr.

[8] Field CB, Behrenfeld MJ, Randerson JT, Falkowski P (1998). Primary production of the biosphere: Integrating terrestrial and oceanic components. Science..

[9] DeVries T, Primeau F, Deutsch C (2012). The sequestration efficiency of the biological pump. Geophys Res Lett.

[10] Cabré A, Marinov I, Leung S (2015). Consistent global responses of marine ecosystems to future climate change across the IPCC AR5 earth system models. Clim Dyn.

[11] Behrenfeld MJ, O’Malley RT, Boss ES, Westberry TK, Graff JR, Halsey KH (2015). Revaluating ocean warming impacts on global phytoplankton. Nat Clim Change.

[12] Richardson K, Bendtsen J (2019). Vertical distribution of phytoplankton and primary production in relation to nutricline depth in the open ocean. Mar Ecol Prog Ser.

[13] Roshan S, DeVries T (2017). Efficient dissolved organic carbon production and export in the oligotrophic ocean. Nat Commun.

[14] Marañón E, Holligan PM, Barciela R, González N, Mouriño B, Pazó MJ (2001). Patterns of phytoplankton size structure and productivity in contrasting open-ocean environments. Mar Ecol Prog Ser.

[15] Pérez V, Fernández E, Marañón E, Morán XAG, Zubkov MV (2006). Vertical distribution of phytoplankton biomass, production and growth in the Atlantic subtropical gyres. Deep Sea Res Part I Oceanographic Res Pap.

[16] Teira E, Mouriño B, Marañón E, Pérez V, Pazó MJ, Serret P (2005). Variability of chlorophyll and primary production in the Eastern North Atlantic subtropical gyre: potential factors affecting phytoplankton activity. Deep Sea Res Part I Oceanographic Res Pap.

[17] Chisholm SW, Frankel SL, Goericke R, Olson RJ, Palenik B, Waterbury JB (1992). *Prochlorococcus marinus* nov. Gen. Nov. Sp.: an oxyphototrophic marine prokaryote containing divinyl chlorophyll *a* and *b*. Arch Microbiol.

[18] Flombaum P, Gallegos JL, Gordillo RA, Rincón J, Zabala LL, Jiao N (2013). Present and future global distributions of the marine cyanobacteria *Prochlorococcus* and *Synechococcus*. PNAS..

[19] Partensky F, Hess WR, Vaulot D (1999). *Prochlorococcus*, a marine photosynthetic prokaryote of global significance. Microbiol Mol Biol Rev.

[20] Li WK (1994). Primary production of prochlorophytes, cyanobacteria, and eucaryotic ultraphytoplankton: Measurements from flow cytometric sorting. Limnol Oceanogr.

[21] Jardillier L, Zubkov MV, Pearman J, Scanlan DJ (2010). Significant CO_2_ fixation by small prymnesiophytes in the subtropical and tropical Northeast Atlantic Ocean. ISME J.

[22] Irion S, Christaki U, Berthelot H, L’Helguen S, Jardillier L (2021). Small phytoplankton contribute greatly to CO_2_-fixation after the diatom bloom in the Southern Ocean. ISME J.

[23] Liu K, Suzuki K, Chen B, Liu H (2020). Are temperature sensitivities of *Prochlorococcus* and *Synechococcus* impacted by nutrient availability in the subtropical Northwest Pacific?. Limnol Oceanogr.

[24] D’Hondt S, Spivack AJ, Pockalny R, Ferdelman TG, Fischer JP, Kallmeyer J (2009). Subseafloor sedimentary life in the South Pacific gyre. PNAS..

[25] Longhurst A, Sathyendranath S, Platt T, Caverhill C (1995). An estimate of global primary production in the ocean from satellite radiometer data. J Plankton Res.

[26] Morel A, Gentili B, Claustre H, Babin M, Bricaud A, Ras J (2007). Optical properties of the “clearest” natural waters. Limnol Oceanogr.

[27] Halm H, Lam P, Ferdelman TG, Lavik G, Dittmar T, LaRoche J (2012). Heterotrophic organisms dominate nitrogen fixation in the south pacific gyre. ISME J.

[28] Raimbault P, Garcia N (2008). Evidence for efficient regenerated production and dinitrogen fixation in nitrogen-deficient waters of the South Pacific Ocean: impact on new and export production estimates. Biogeosciences..

[29] Shiozaki T, Bombar D, Riemann L, Sato M, Hashihama F, Kodama T (2018). Linkage between dinitrogen fixation and primary production in the oligotrophic South Pacific Ocean. Glob Biogeochem Cyc.

[30] Reintjes G, Tegetmeyer HE, Bürgisser M, Orlić S, Tews I, Zubkov M (2019). On-site analysis of bacterial communities of the ultraoligotrophic South Pacific gyre. Appl Environ Microbiol.

[31] Zielinski O, Henkel R, Voß D, Ferdelman TG. Physical oceanography during Sonne cruise SO245 (Ultrapac)*.* PANGAEA. 2018. 10.1594/PANGAEA.890394.

[32] Ferdelman TG, Klockgether G, Downes P, Lavik G. Nutrient data from CTD Nisken bottles from Sonne expedition SO-245 “Ultrapac”. PANGAEA. 2019. 10.1594/PANGAEA.899228.

[33] Arar EJ, Collins GB. Method 445.0: In vitro determination of chlorophyll *a* and pheophytin *a* in marine and freshwater algae by fluorescence: U.S. Environmental Protection Agency, Washington, DC; 1997. https://cfpub.epa.gov/si/si_public_record_report.cfm?Lab=NERL&dirEntryId=309417.

[34] Welschmeyer N, Naughton S (1994). Improved chlorophyll *a* analysis: single fluorometric measurement with no acidification. Lake Reserv Manag.

[35] Osterholz H, Kilgour D, Storey DS, Lavik G, Ferdelman T, Niggemann J (2021). Accumulation of DOC in the South Pacific subtropical gyre from a molecular perspective. Mar Chem.

[36] Voß D, Henkel R, Wollschläger J, Zielinski O. Hyperspectral underwater light field measured during the cruise SO245 with R/V Sonne. PANGAEA. 2020. 10.1594/PANGAEA.911558.

[37] Martínez-Pérez C, Mohr W, Löscher CR, Dekaezemacker J, Littmann S, Yilmaz P (2016). The small unicellular diazotrophic symbiont, UCYN-A, is a key player in the marine nitrogen cycle. Nat Microbiol..

[38] Marra J (2009). Net and gross productivity: weighing in with ^14^C. Aquat Microb Ecol.

[39] Ribeiro CG, Marie D, Santos ALD, Brandini FP, Vaulot D (2016). Estimating microbial populations by flow cytometry: comparison between instruments. Limnol Oceanogr Methods.

[40] Pernthaler A, Pernthaler J, Amann R (2002). Fluorescence in situ hybridization and catalyzed reporter deposition for the identification of marine bacteria. Appl Environ Microbiol.

[41] West NJ, Schönhuber WA, Fuller NJ, Amann RI, Rippka R, Post AF (2001). Closely related *Prochlorococcus* genotypes show remarkably different depth distributions in two oceanic regions as revealed by in situ hybridization using 16 S rRNA-targeted oligonucleotides. Microbiology..

[42] Polerecky L, Adam B, Milucka J, Musat N, Vagner T, Kuypers MMM (2012). Look@NanoSIMS—a tool for the analysis of nanoSIMS data in environmental microbiology. Environ Microbiol.

[43] Verity PG, Robertson CY, Tronzo CR, Andrews MG, Nelson JR, Sieracki ME (1992). Relationships between cell volume and the carbon and nitrogen content of marine photosynthetic nanoplankton. Limnol Oceanogr.

[44] Khachikyan A, Milucka J, Littmann S, Ahmerkamp S, Meador T, Könneke M (2019). Direct cell mass measurements expand the role of small microorganisms in nature. Appl Environ Microbiol.

[45] Walters W, Hyde ER, Berg-Lyons D, Ackermann G, Humphrey G, Parada A (2016). Improved bacterial 16 S rRNA gene (v4 and v4-5) and fungal internal transcribed spacer marker gene primers for microbial community surveys. MSystems.

[46] Parada AE, Needham DM, Fuhrman JA (2016). Every base matters: assessing small subunit rrna primers for marine microbiomes with mock communities, time series and global field samples. Environ Microbiol.

[47] Comeau AM, Douglas GM, Langille MG (2017). Microbiome helper: a custom and streamlined workflow for microbiome research. MSystems.

[48] Caporaso JG, Kuczynski J, Stombaugh J, Bittinger K, Bushman FD, Costello EK (2010). Qiime allows analysis of high-throughput community sequencing data. Nat Methods.

[49] Haas S, Desai DK, LaRoche J, Pawlowicz R, Wallace DW (2019). Geomicrobiology of the carbon, nitrogen and sulphur cycles in Powell Lake: a permanently stratified water column containing ancient seawater. Environ Microbiol.

[50] Zhang J, Kobert K, Flouri T, Stamatakis A. Pear: a fast and accurate Illumina paired-end read merger. Bioinformatics. 2013;30:614–20. 10.1093/bioinformatics/btt593 PMC3933873 24142950

[51] Rognes T, Flouri T, Nichols B, Quince C, Mahé F (2016). Vsearch: a versatile open source tool for metagenomics. PeerJ..

[52] Kopylova E, Noé L, Touzet H (2012). Sortmerna: fast and accurate filtering of ribosomal RNAs in metatranscriptomic data. Bioinformatics..

[53] Mercier C, Boyer F, Bonin A, Coissac E (eds). Sumatra and Sumaclust: fast and exact comparison and clustering of sequences. SeqBio 2013 Workshop 2013: (abstract).

[54] DeSantis TZ, Hugenholtz P, Larsen N, Rojas M, Brodie EL, Keller K (2006). Greengenes, a chimera-checked 16 S rRNA gene database and workbench compatible with ARB. Appl Environ Microbiol.

[55] Quast C, Pruesse E, Yilmaz P, Gerken J, Schweer T, Yarza P (2012). The SILVA ribosomal RNA gene database project: improved data processing and web-based tools. Nucleic Acids Res.

[56] Decelle J, Romac S, Stern RF, Bendif EM, Zingone A, Audic S (2015). PhytoREF: A reference database of the plastidial 16 S rRNA gene of photosynthetic eukaryotes with curated taxonomy. Molec Ecol Res..

[57] Guillou L, Bachar D, Audic S, Bass D, Berney C, Bittner L (2012). The protist ribosomal reference database (PR2): a catalog of unicellular eukaryote small sub-unit rRNA sequences with curated taxonomy. Nucleic Acids Res.

[58] Del Campo J, Kolisko M, Boscaro V, Santoferrara LF, Nenarokov S, Massana R (2018). EukRef: phylogenetic curation of ribosomal RNA to enhance understanding of eukaryotic diversity and distribution. PLoS Biol.

[59] Ludwig W, Strunk O, Westram R, Richter L, Meier H, Yadhukumar (2004). ARB: A software environment for sequence data. Nucleic Acids Res.

[60] Gruber-Vodicka HR, Seah BK, Pruesse E (2020). Phyloflash: rapid small-subunit rRNA profiling and targeted assembly from metagenomes. Msystems..

[61] Farrant GK, Doré H, Cornejo-Castillo FM, Partensky F, Ratin M, Ostrowski M (2016). Delineating ecologically significant taxonomic units from global patterns of marine picocyanobacteria. PNAS.

[62] Oggerin de Orube M, Fuchs BM. Personal communication: Unpublished shotgun metagenomes collected from in situ pump samples during R/V Sonne expedition SO245. Bremen, Germany. 2021.

[63] Schlitzer R. Ocean Data View. Bremerhaven, Germany. 2021. https://odv.awi.de.

[64] R Core Team. R: A language and environment for statistical computing. R Foundation for Statistical Computing,Vienna, Austria. 2017. https://www.R-project.org/.

[65] Wickham H. Ggplot2: elegant graphics for data analysis. Springer-Verlag, New York. 2016.

[66] McMurdie PJ, Holmes S (2013). Phyloseq: an R package for reproducible interactive analysis and graphics of microbiome census data. PLOS ONE.

[67] Oksanen J, Kindt R, Legendre P, O’Hara B, Stevens MHH, Oksanen MJ, et al. The vegan package: community ecology package. R package version 2.5–7. 2019. https://CRAN.R-project.org/package=vegan.

[68] Chaigneau A, Pizarro O. Surface circulation and fronts of the South Pacific Ocean, east of 120°W. Geophys Res Lett. 2005;32:L08605.

[69] Logares R, Sunagawa S, Salazar G, Cornejo‐Castillo FM, Ferrera I, Sarmento H (2014). Metagenomic 16 S rRNA Illumina tags are a powerful alternative to amplicon sequencing to explore diversity and structure of microbial communities. Environ Microbiol.

[70] Shi XL, Lepère C, Scanlan DJ, Vaulot D (2011). Plastid 16 s rRNA gene diversity among eukaryotic picophytoplankton sorted by flow cytometry from the South Pacific Ocean. PLOS ONE.

[71] Fuller NJ, Campbell C, Allen DJ, Pitt FD, Zwirglmaier K, Le Gall F (2006). Analysis of photosynthetic picoeukaryote diversity at open ocean sites in the Arabian Sea using a pcr biased towards marine algal plastids. Aquat Micro Ecol.

[72] Raes EJ, Bodrossy L, Kamp JVD, Bissett A, Ostrowski M, Brown MV (2018). Oceanographic boundaries constrain microbial diversity gradients in the South Pacific Ocean. PNAS.

[73] Campbell L, Liu H, Nolla HA, Vaulot D (1997). Annual variability of phytoplankton and bacteria in the subtropical North Pacific Ocean at station ALOHA during the 1991-4 ENSO event. Deep Sea Res Part I Oceanogr Res Pap.

[74] Viviani DA, Church MJ (2017). Decoupling between bacterial production and primary production over multiple time scales in the North Pacific subtropical gyre. Deep Sea Res Part I Oceanogr Res Pap.

[75] Rii YM, Duhamel S, Bidigare RR, Karl DM, Repeta DJ, Church MJ (2016). Diversity and productivity of photosynthetic picoeukaryotes in biogeochemically distinct regions of the south east pacific ocean. Limnol Oceanogr.

[76] Shi XL, Marie D, Jardillier L, Scanlan DJ, Vaulot D (2009). Groups without cultured representatives dominate eukaryotic picophytoplankton in the oligotrophic South East Pacific Ocean. PLOS ONE.

[77] Kirkham AR, Lepere C, Jardillier LE, Not F, Bouman H, Mead A (2013). A global perspective on marine photosynthetic picoeukaryote community structure. ISME J.

[78] Lepère C, Vaulot D, Scanlan DJ (2009). Photosynthetic picoeukaryote community structure in the South East Pacific Ocean encompassing the most oligotrophic waters on earth. Environ Microbiol.

[79] Bender ML, Jönsson B (2016). Is seasonal net community production in the South Pacific subtropical gyre anomalously low?. Geophys Res Lett.

[80] Montégut CDB, Madec G, Fischer AS, Lazar A, Iudicone D (2004). Mixed layer depth over the global ocean: an examination of profile data and a profile-based climatology. J Geophys Res Oceans.

[81] Liu Q, Lu Y (2016). Role of horizontal density advection in seasonal deepening of the mixed layer in the subtropical Southeast Pacific. Adv Atmospher Sci.

[82] Sato K, Suga T (2009). Structure and modification of the South Pacific eastern subtropical mode water. J Phys Oceanogr.

[83] Jung J, Furutani H, Uematsu M (2011). Atmospheric inorganic nitrogen in marine aerosol and precipitation and its deposition to the north and south pacific oceans. J Atmospher Chem..

[84] Pavia FJ, Anderson RF, Winckler G, Fleisher MQ (2020). Atmospheric dust inputs, iron cycling, and biogeochemical connections in the South Pacific Ocean from thorium isotopes. Glob Biogeochem Cycles.

[85] Bonnet S, Guieu C, Bruyant F, Prášil O, Van Wambeke F, Raimbault P (2008). Nutrient limitation of primary productivity in the Southeast Pacific (Biosope Cruise). Biogeosciences..

[86] Mahaffey C, Björkman KM, Karl DM (2012). Phytoplankton response to deep seawater nutrient addition in the North Pacific subtropical gyre. Mar Ecol Prog Ser.

[87] Grob C, Jardillier L, Hartmann M, Ostrowski M, Zubkov MV, Scanlan DJ (2015). Cell-specific CO_2_ fixation rates of two distinct groups of plastidic protists in the Atlantic Ocean remain unchanged after nutrient addition. Environ Microbiol Rep.

[88] Vaulot D, Marie D, Olson RJ, Chisholm SW (1995). Growth of *Prochlorococcus*, a photosynthetic prokaryote, in the equatorial pacific ocean. Science..

[89] Grob C, Hartmann M, Zubkov MV, Scanlan DJ (2011). Invariable biomass-specific primary production of taxonomically discrete picoeukaryote groups across the Atlantic Ocean. Environ Microbiol.

[90] Berthelot H, Duhamel S, L’Helguen S, Maguer J-F, Wang S, Cetinić I (2019). NanoSIMS single cell analyses reveal the contrasting nitrogen sources for small phytoplankton. ISME J.

[91] Zubkov MV, Fuchs BM, Tarran GA, Burkill PH, Amann R (2003). High rate of uptake of organic nitrogen compounds by *Prochlorococcus* cyanobacteria as a key to their dominance in oligotrophic oceanic waters. Appl Environ Microbiol.

[92] Muñoz-Marín MC, Gómez-Baena G, López-Lozano A, Moreno-Cabezuelo JA, Díez J, García-Fernández JM (2020). Mixotrophy in marine picocyanobacteria: use of organic compounds by *Prochlorococcus* and *Synechococcus*. ISME J.

[93] Timmermans K, Van der Wagt B, Veldhuis M, Maatman A, De Baar H (2005). Physiological responses of three species of marine pico-phytoplankton to ammonium, phosphate, iron and light limitation. J Sea Res.

[94] Vaulot D, Eikrem W, Viprey M, Moreau H (2008). The diversity of small eukaryotic phytoplankton (≤ 3 μm) in marine ecosystems. FEMS Microbiol Rev.

[95] Worden AZ, Janouskovec J, McRose D, Engman A, Welsh RM, Malfatti S (2012). Global distribution of a wild alga revealed by targeted metagenomics. Curr Biol.

[96] Le Gall F, Rigaut-Jalabert F, Marie D, Garczarek L, Viprey M, Gobet A (2008). Picoplankton diversity in the South-east Pacific Ocean from cultures. Biogeosciences..

[97] NASA Goddard Space Flight Center, Ocean Ecology Laboratory, Ocean Biology Processing Group. Moderate-resolution Imaging Spectroradiometer (MODIS) Aqua Chlorophyll Data; Reprocessing. NASA OB.DAAC, Greenbelt, MD, USA. 2018. https://oceancolor.gsfc.nasa.gov/data/10.5067/AQUA/MODIS/L3M/CHL/2018/ Accessed 2019/08/01.

