# Supplementary material for: Niche partitioning by photosynthetic plankton as a driver of CO2-fixation across the oligotrophic South Pacific Subtropical Ocean
Source: ISME J. 2021 Aug 19;16(2):465–76. doi: 10.1038/s41396-021-01072-z (PMC8776750; doi:10.1038/s41396-021-01072-z)
Supplement: Supplementary file 1 — Supplemental File 1: Supplementary Information [file 41396_2021_1072_MOESM1_ESM.pdf]

Supplemental File 1

Supplementary Information to

Niche partitioning by photosynthetic plankton as a driver of CO_2-fixation across the oligotrophic South Pacific Sub-tropical Ocean

Duerschlag et al.; The ISME Journal, 2021

This Supplementary Information file (Supplemental File 1) contains:

I. Supplementary Tables (2 tables)
II. Supplementary Figures (19 Figures)
III. Extended description of the materials and methods employed in the study.
IV. References Cited in Supplementary Information

I. Supplementary Tables

Table S1. Data Deposition

Data	Location and Link/Accession Number
Light field	*Pangaea:* https://doi.org/10.1594/PANGAEA.911558
Salinity-Temperature (CTD)	*Pangaea:* https://doi.org/10.1594/PANGAEA.890394
Nutrients	*Pangaea:* https://doi.pangaea.de/10.1594/PANGAEA.899228
Supplemental Data Tables<br>-Station List & CO_2 fixation rates<br>-Single Cell Rates<br>-Figure 7 Statistics	*MPG Edmond Data Repository*:<br>https://edmond.mpdl.mpg.de/imeji/collection/_rDo5ch6hHYOCwfN<br>https://dx.doi.org/10.17617/3.6q
Flow Cytometry data:	*Flow Repository*:<br>https://flowrepository.org/id/RvFrru2M2yxF4dtwRMw1hAgFPMmQdqgd9Ki3Kamo4FWdAhzjjCkAhAR6sapm3c6Q
Metabarcoding scripts	https://github.com/LangilleLab/microbiome_helper/wiki/16S-Bacteria-and-Archaea-Standard-Operating-Procedure
Metabarcodes and Gene Read Sequences	*NCBI Sequence Read Archive [SRA] under Bioproject: PRJNA670604*<br>https://www.ncbi.nlm.nih.gov/bioproject/
-16S rRNA Amplicon Metabarcodes	PRJNA670604
-Chloroplast Metabarcodes	MW152420-MW153037 *Nucleotide Codes*
-Metagenome 18S rRNA metabarcodes	PRJNA670604
-Metagenome 16S rRNA metabarcodes	PRJNA670604
-Metagenomic petB gene read sequences	PRJNA670604

Table S2. Summary of single-cell rates for *Prochlorococcus* and small photosynthetic eukaryotes.

	oligotrophic SPG				mesotrophic SWP	
**Station**	**4**	**4**	**8**	**8**	**12**	**12**
**Category**	surface	chl *a* max	surface	chl a max	surface	chl a max
**Depth [m]**	22	178	21	182	21	101
**chl a [µg l^{-1}]**	0.03	0.53	<0.01	0.65	0.49	0.78
**Abundance [cells 10^{6} l^{-1}]*,****						
**Small eukaryotes**	0.31 ± 0.03 (11-40)	0.83 ± 0.21 (150-225)	0.56 ± 0.04 (5-40)	1.66 ± 0.4 (151-200)	5.5 ± 0.3 (6-41)	2.1 ± 0.7 (61-150)
Prochlorococcus	70.6 ± 3.5 (11-40)	88.1 ± 41.9 (150-225)	35.9 ± 8.2 (5-40)	58.2 ± 7.2 (151-200)	72.4 ± 20.2 (11-41)	37.9 ± 5.8 (61-150)
**Size (length) range of measured cells [µm]***						
**Small eukaryotes**	1.5-4.6 (2.1 ± 0.1) n=25	1.5-3.6 (1.9 ±0.1) n=24	1.5-4.8 (2.4 ± 0.1) n=34	1.6-3.5 (1.8 ± 0.1) n=23	1.7-4.1 (2.6 ± 0.1) n=31	1.4-3.9 (2.1 ± 0.1) n=28
Prochlorococcus	0.3-0.7 (0.5 ±0.02) n=26	0.5-0.9 (0.6 ±0.02) n=59	0.3-0.9 (0.5 ± 0.02) n=56	0.4-0.8 (0.5 ± 0.02) n=28	0.4-1.1 (0.6 ± 0.04) n=25	0.3-0.8 (0.6 ± 0.02) n=55
**Cell-specific CO_2 fixation rates [fmol C cell^{-1} d^{-1}]***						
**Small eukaryotes**	14.1 ± 2.3	15.6 ± 3.0	15.8 ± 4.0	15.6 ± 2.3	50.3 ± 7.9	23.4 ± 6.3
Prochlorococcus	0.26 ±	0.52 ± 0.08	0.17 ± 0.02	0.31 ± 0.04	0.6 ± 0.2	0.46 ± 0.05
**C-based growth rate [d^{-1}] ***						
**Small eukaryotes**	0.17 ± 0.02	0.39 ± 0.02	0.16 ± 0.01	0.33 ± 0.02	0.56 ± 0.03	0.42 ± 0.01
Prochlorococcus	0.13 ± 0.02	0.16 ± 0.02	0.06 ± 0.01	0.08 ± 0.01	0.2 ± 0.03	0.13 ± 0.01
**Contribution to bulk C fixation rate [µmol C l^{-1} d^{-1}]*,*****						
**Small eukaryotes**	0.004 ± 0.001 (7.7%)	0.01 ± 0.004 (12.4%)	0.01 ± 0.002 (11%)	0.03 ± 0.007 (21%)	0.28 ± 0.05 (38.4%)	0.05 ± 0.02 (24%)
Prochlorococcus	0.02 ± 0.004 (32%)	0.05 ± 0.02 (44.4%)	0.006 ± 0.002 (7.5%)	0.02 ± 0.003 (14.9%)	0.04 ± 0.02 (6%)	0.02 ± 0.003 (8.3%)

* values are median ± standard error ** values in brackets represent depth range in m used to determine cell abundance via FCM. *** median values used for contribution calculations

II. Supplementary Figures

a

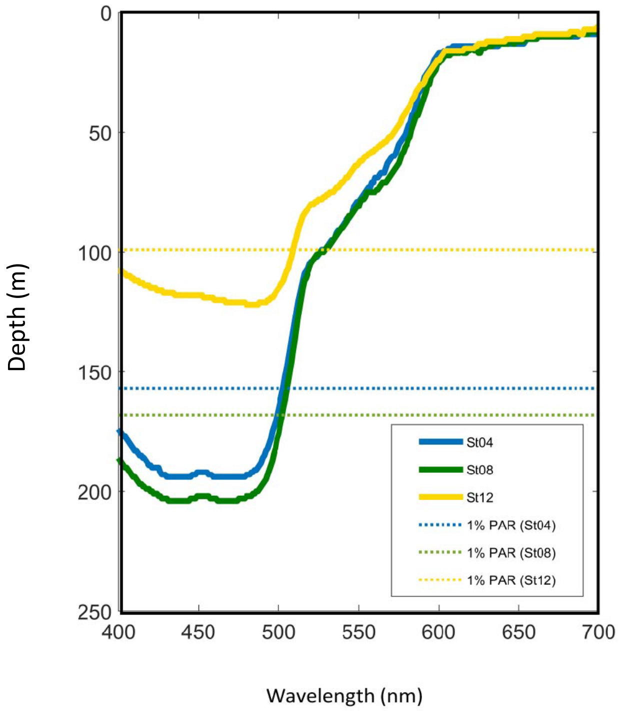

b.

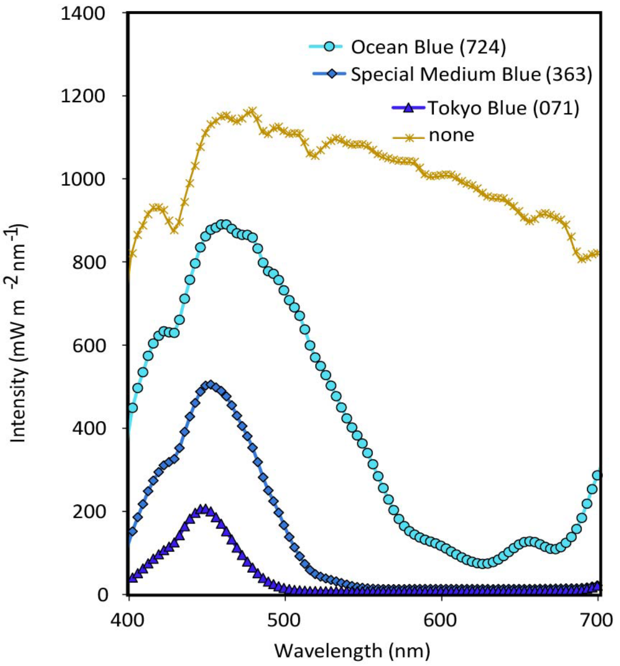

Figure S1: Light spectra in the water column and in onboard incubation samples used for CO_2 fixation experiments: a. Spectral penetration depths (1%), and 1% total photosynthetic available radiation (PAR) for three stations 4 (blue), 8 (green) and 12 (yellow). **b.** Measured spectral intensity for the three filters used in onboard incubators (Ocean Blue = 33% PAR; Special Medium Blue = 4.2% PAR; Tokyo Blue = 1% PAR).

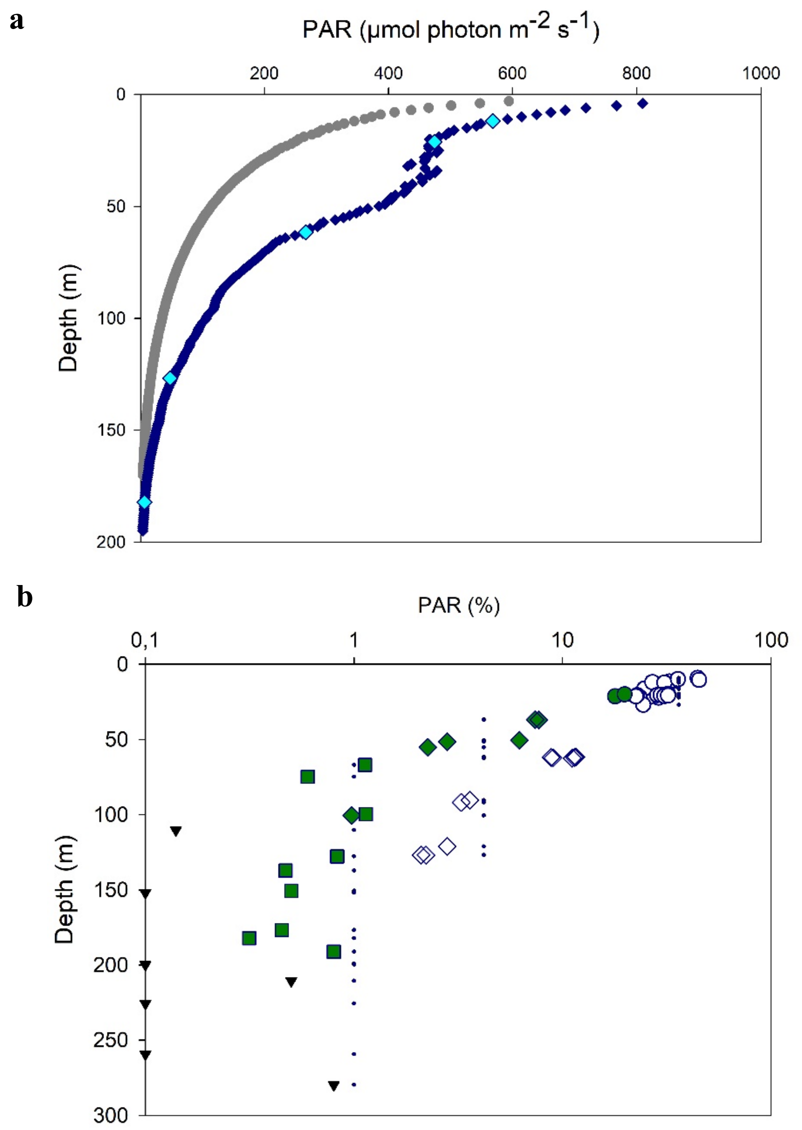

Figure S2: Photosynthetic available radiation (PAR) in the water column and in onboard incubation samples used for CO_2 fixation experiments:

a. PAR measured at midday at Station SO245-08 on two consecutive days, Jan 7. 2016 (gray circles) and Jan. 8, 2016 (blue circles). Open light blue diamonds show the expected PAR values experienced at mid-day by CO_2 fixation experiments performed on January 8, 2016. Incident radiation at the surface can vary up to two-fold. During the noon-time PAR measurements on January 8, shipboard measurements of incident global radiation were 400 Wm^{-2}, but ranged from values of 250 to 680 W m^{-2} during the course of the day (midday average 480 W m^{-2}). Wave effects on sunny days can lead to focusing effects as seen in the sunny profile (blue) at S1d (e.g. constant or increasing values at 30-40 meters), whereas such effects are absent on cloudy, diffuse days (gray profile).

b. %PAR experienced by samples in the onboard incubators. Black dots represent the %PAR cutoffs for the three filters, and corresponding samples are note with circles (Ocean Blue 36.1%), diamonds (Special Medium Blue 4.2%), and squares (Tokyo Blue 1%). Open symbols show samples from the surface and above the chl a max, closed dark green symbols are for samples from within the chl a max (>0.5 ug l-1 as shown in Figure 1b). Dark triangles represent samples from below the chl *a* max (incubated at 1%PAR).

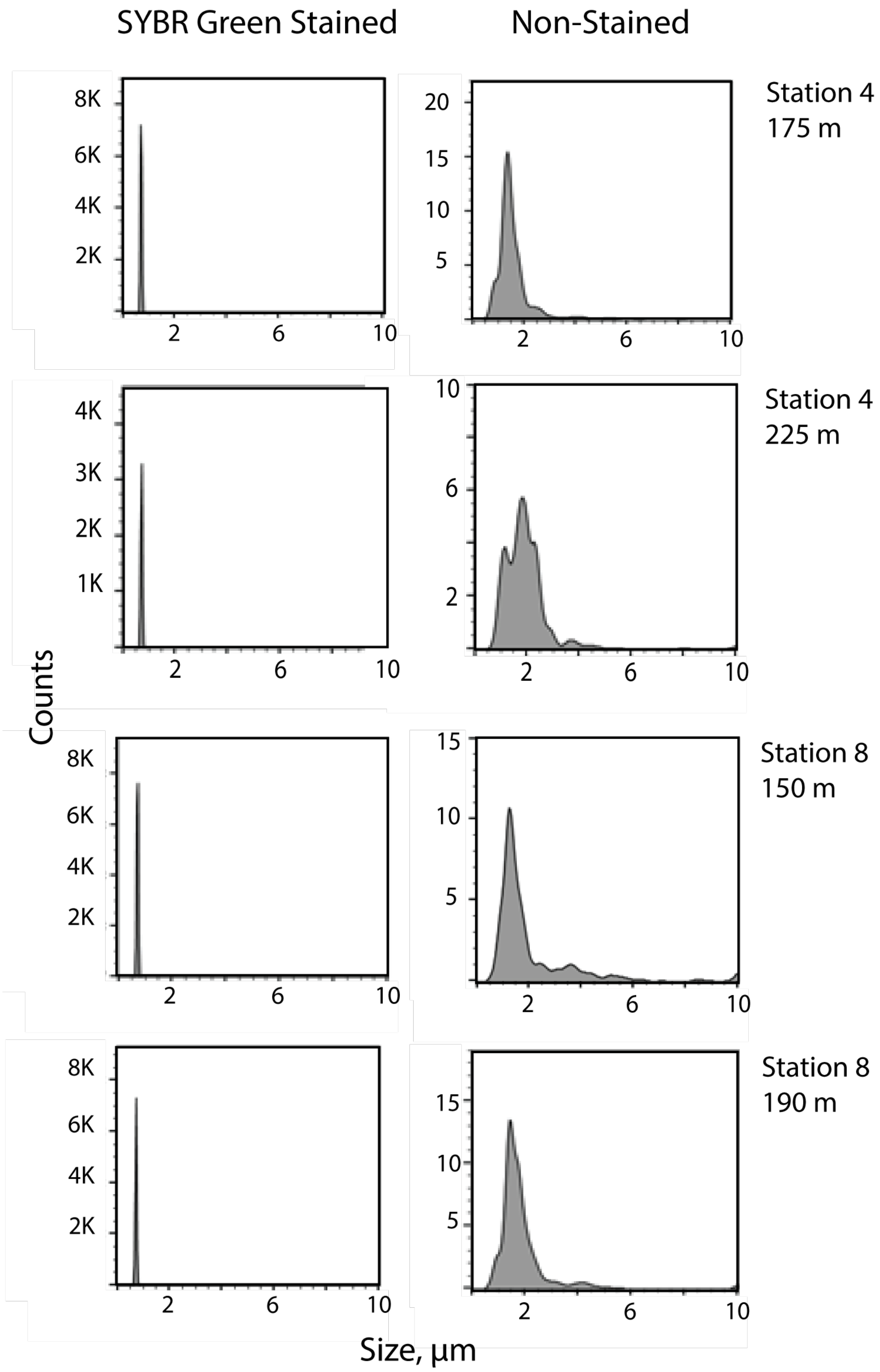

Figure S3: Example size distributions obtained using Flow Cytometry from near the deep chl *a* maximum of Stations 4 and 8. The left-hand panels show *Prochlorococcus* after SYBR-Green staining and the right-hand panels show the pico-eukaryote fraction in the non-stained samples on forward scatter (FSC).

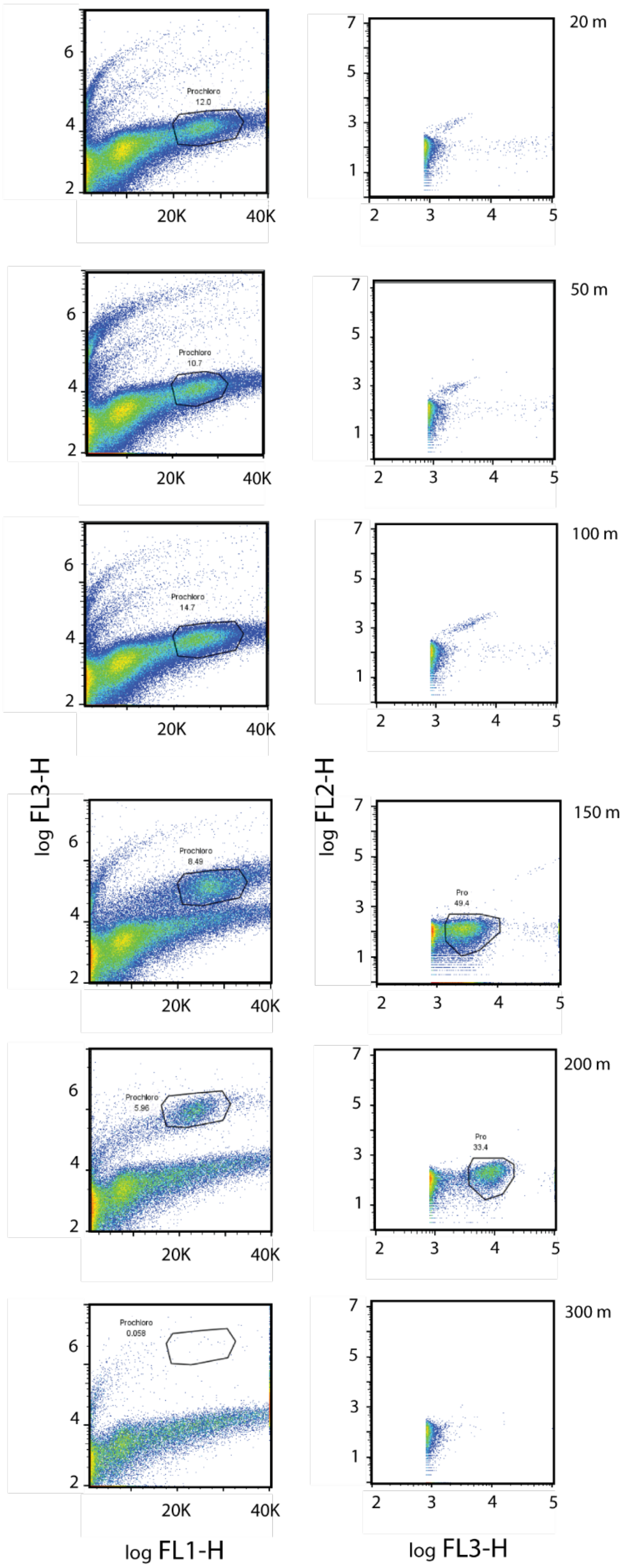

Figure S4: Flow cytograms showing (left-hand panels) FL1 versus FL3 for SYBR Green stained cells and (right-hand panels) FL2 versus FL3 for unstained cells at Station 3.

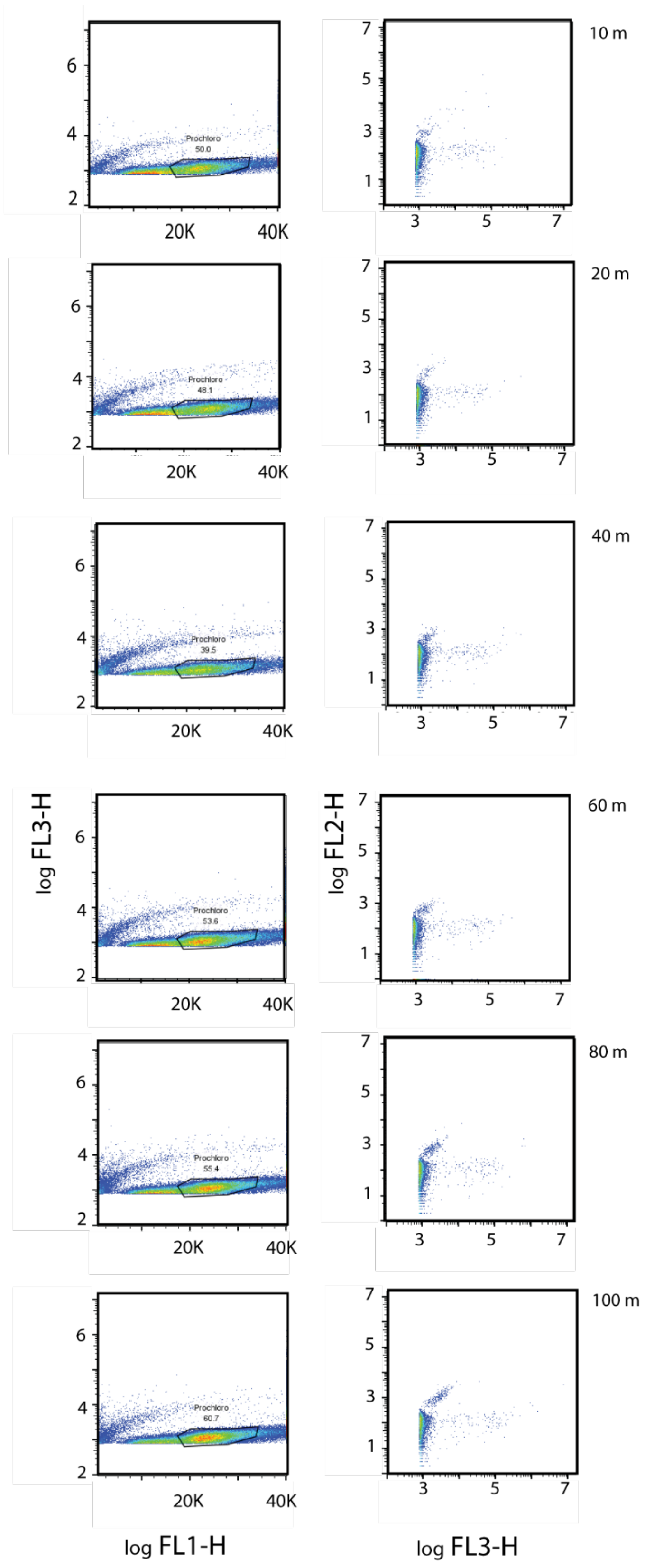

S5: Flow cytograms showing (left-hand panels) FL1 versus FL3 for SYBR Green stained cells and (right-hand panels) FL2 versus FL3 for unstained cells in the upper 100 meters of Station 4.

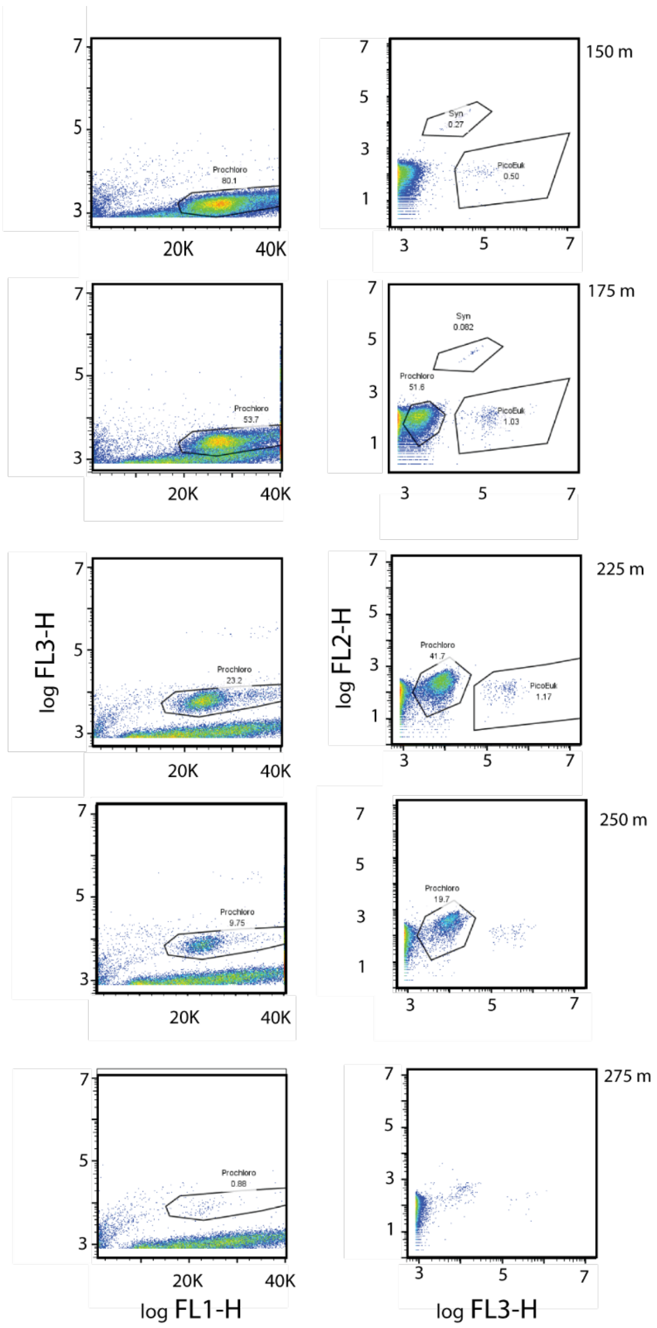

S6: Flow cytograms (left-hand panels) FL1 versus FL3 for SYBR Green stained cells and (right-hand panels) FL2 versus FL3 for unstained cells in 125 to 275 m of Station 4.

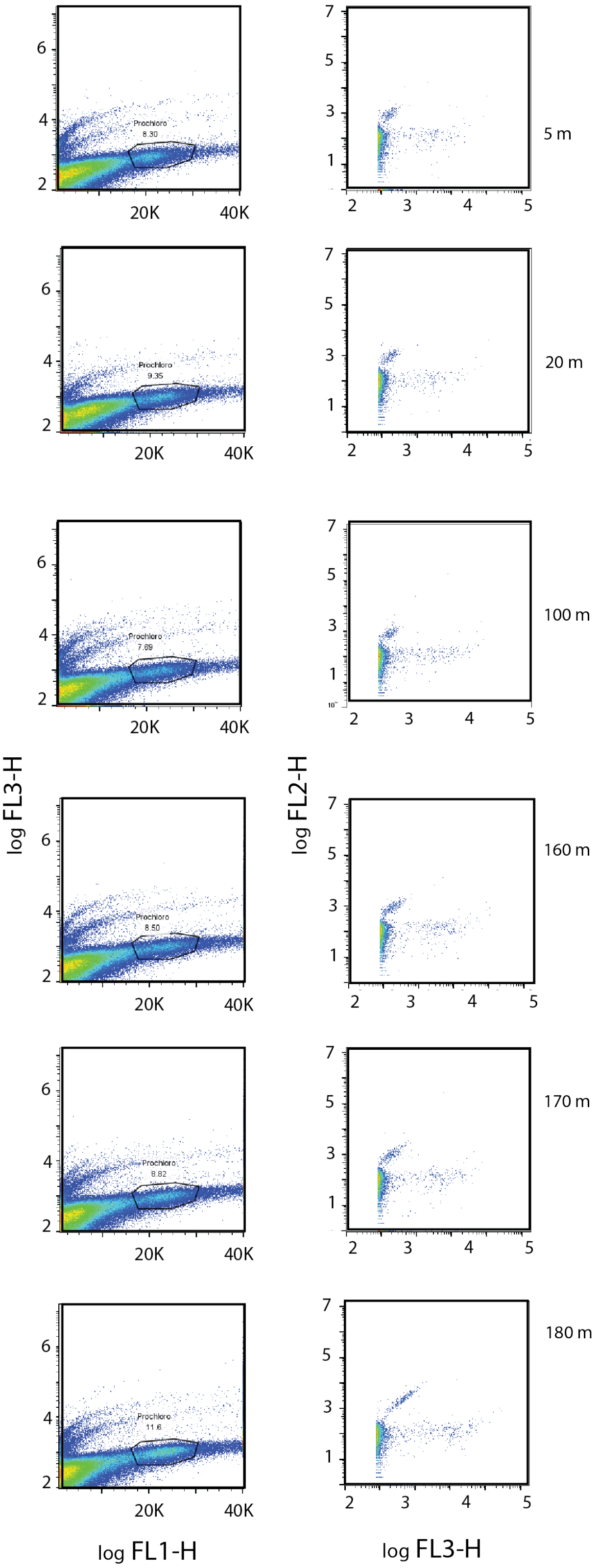

S7: Flow cytograms showing (left-hand panels) FL1 versus FL3 for SYBR Green stained cells and (right-hand panels) FL2 versus FL3 for unstained cells in the upper 180 meters of Station 8.

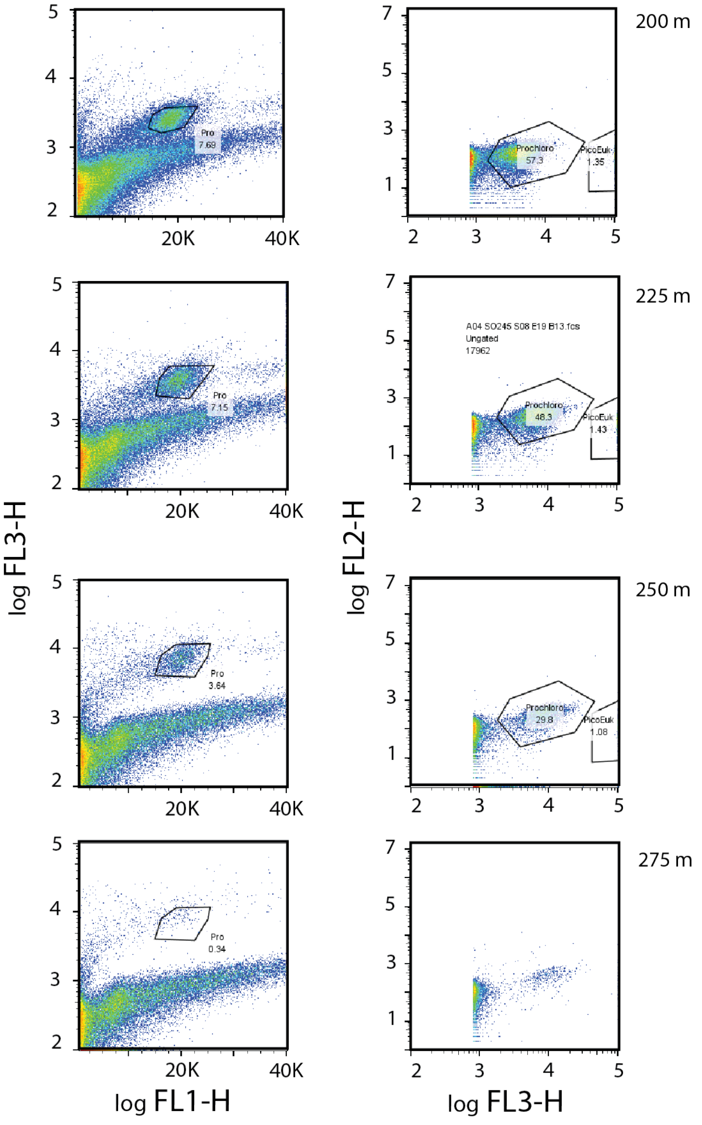

S8: Flow cytograms showing (left-hand panels) FL1 versus FL3 for SYBR Green stained cells and (right-hand panels) FL2 versus FL3 for unstained cells in 150 to 275 meters of Station 8.

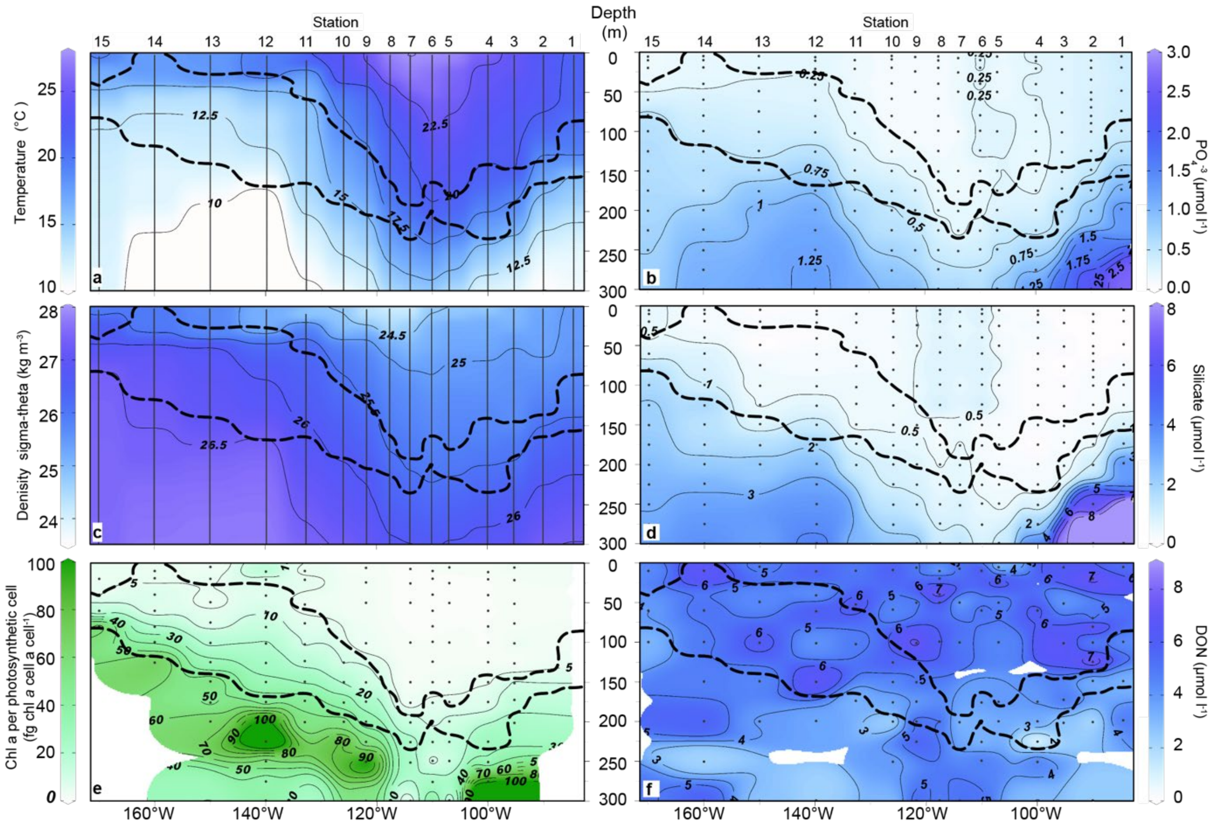

Figure S9: Distribution of (a) temperature, (b) phosphate (PO_4^{3-}) concentration, (c) density sigma-theta, (d) silicate concentration, (e) chl *a* per photosynthetic cell and dissolved organic nitrogen (DON) of the upper 300 m of the South Pacific Gyre. Black dots mark the individual samples or measurement. The thick dashed line represents the 0.5 µg L^{-1} chl *a* isoline and illustrates the chl *a* max.

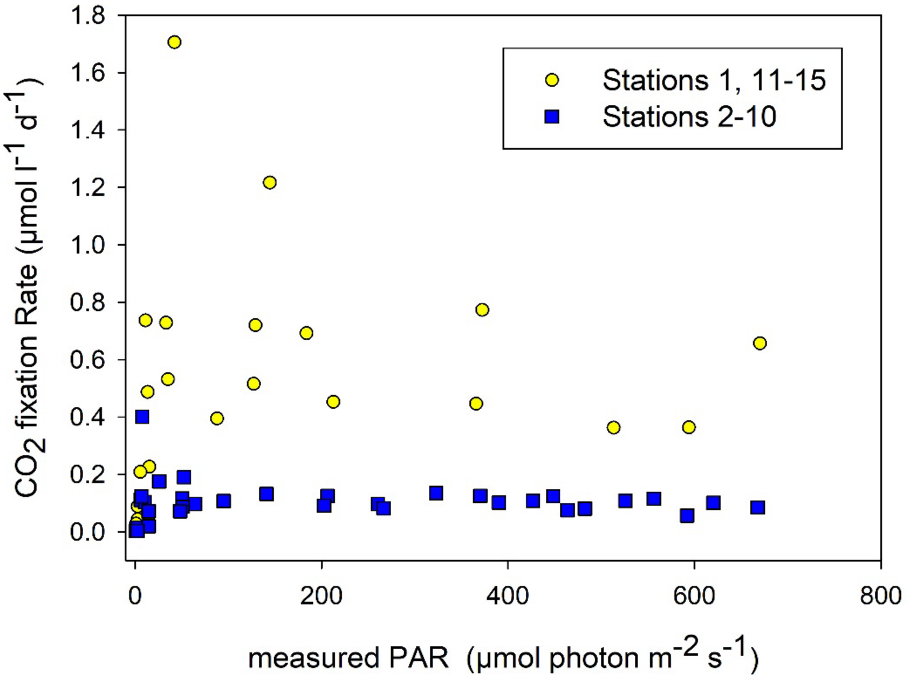

Figure S10: CO_2 fixation rates as a function of measured photosynthetically available radiation (PAR). Squares show data from the SPG (Stations 2-10) and circles from the Eastern South Pacific (Station 1) and Southwest Pacific (Stations 11-15).

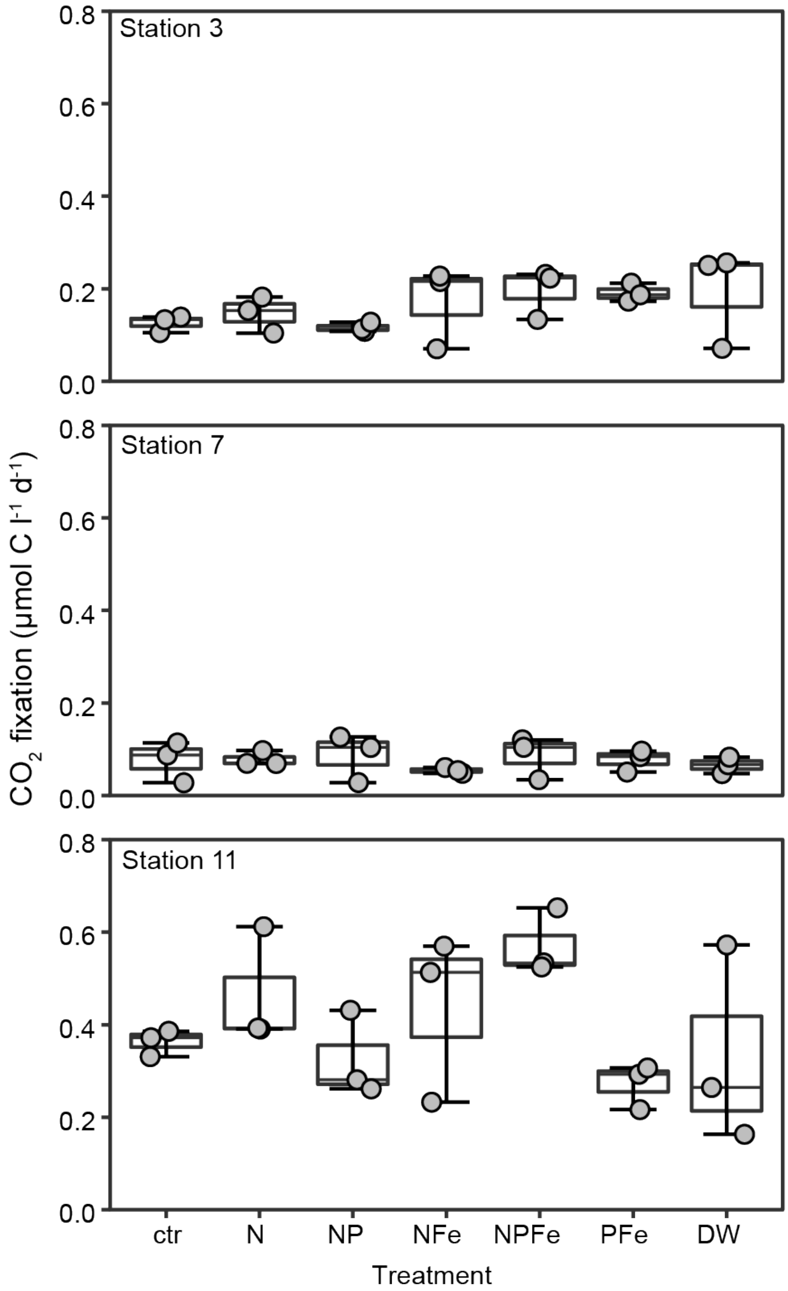

Figure S11. Nutrient manipulation experiments. CO_2 fixation rates from nutrient addition 24 hour experiments on samples amended with various combinations of nutrients, where: ctr (control without any addition), N (addition of $(NH_4)_2SO_4$ and $NaNO_3$), P (addition of NaH_2PO_4), Fe ($FeCl_2$), and deep water (DW) obtained from 2500 m water depth. The error bars represent the standard deviation from 24 hour triplicate incubations on samples obtained from 20 meter water depth. Treatments were compared using one-way ANOVA and a Tukey multiple pairwise comparison test. Means that are significantly different from the control are labeled with an asterisk (*p<0.05*). None of these treatments were significantly different to the control.

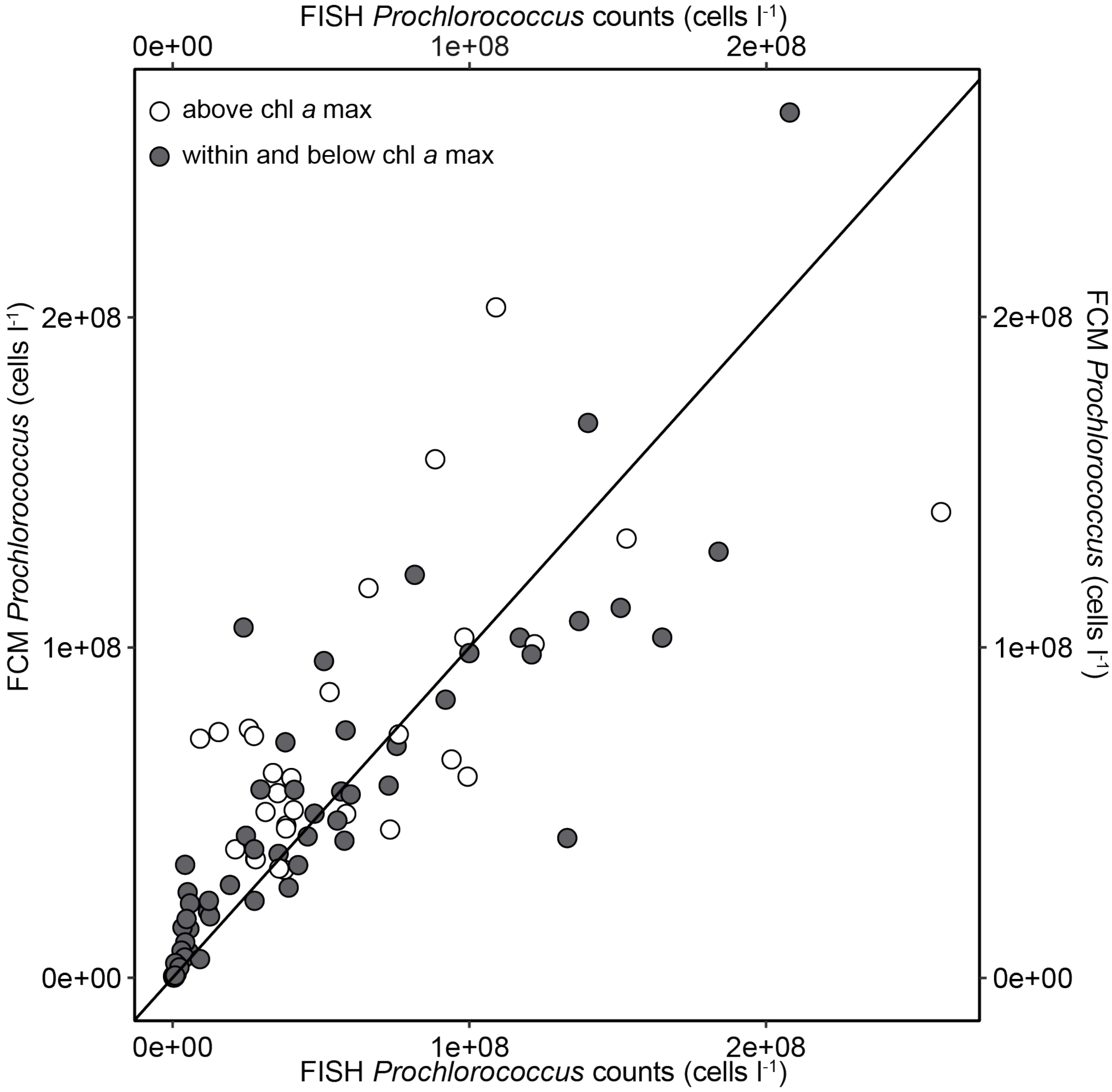

Figure S12: Comparison of flow cytometry estimates of *Prochlorococcus* versus estimates from Fluorescent In Situ Hybridization counts (FISH) taken (2). FISH data is from Reintjes et al., 2019 (2) obtained contemporaneously during Expedition SO245. Open circles are from above the chl *a* max and dark circles are from within or below the chl *a* max.

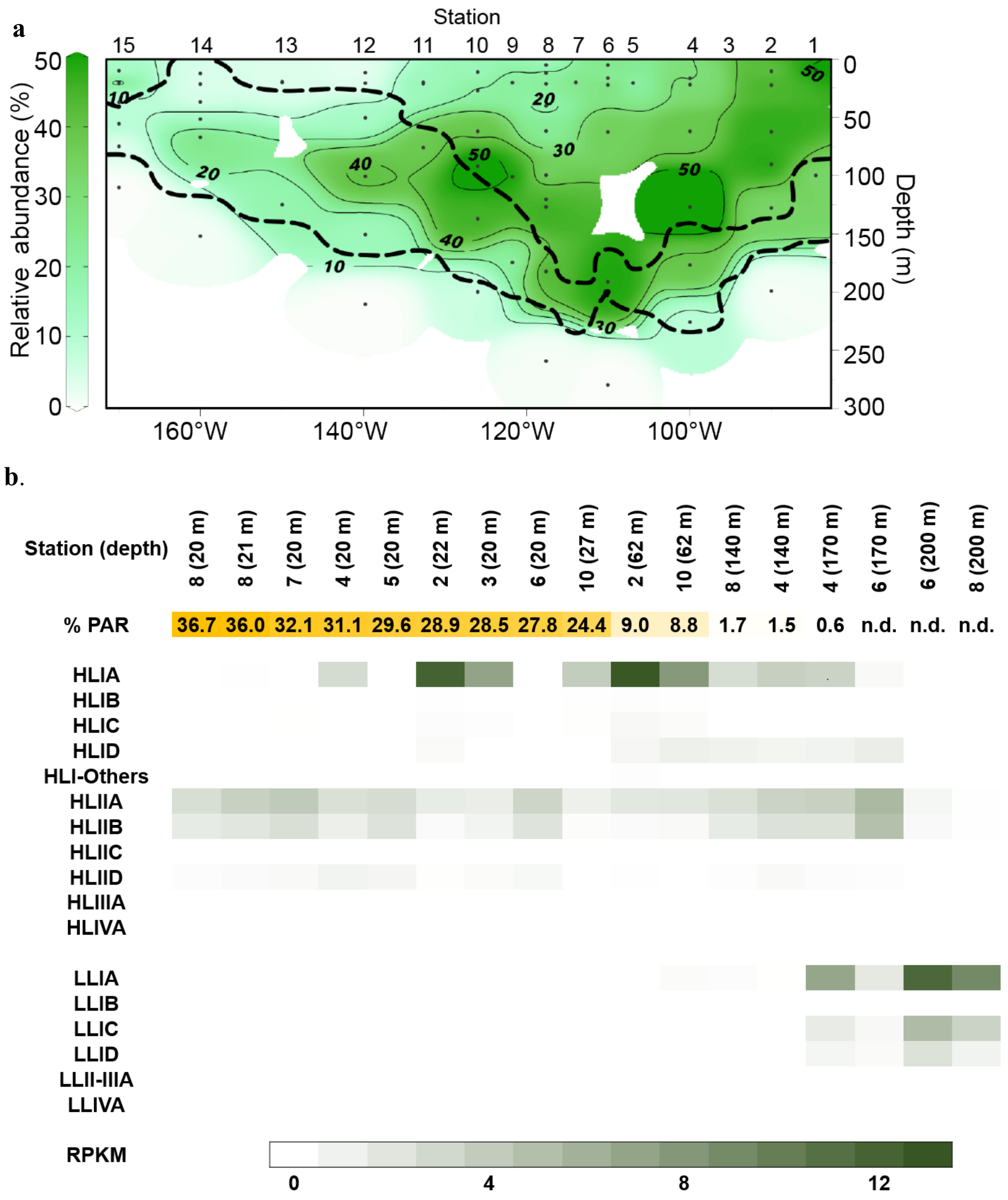

Figure S13: Relative abundances of *Prochlorococcus* 16S rRNA and petB genes. (a). Distribution of 16S rRNA relative OTU abundance [%] of total number of sequences recovered from each station/depth (total 16S rRNA dataset rarefied to 15000 reads per sample) of all *Prochlorococcus* Black dots mark the individual samples. The thick dashed line represents the 0.5 µg l^{-1} chl *a* isoline and illustrates the chl *a* max. **(b)** Heat map of relative petB gene read (3) distribution as a function of %PAR. Calculated Reads per kilobase per 10^6 mapped reads (RPKM) of all entries were summed within each ecologically significant taxonomic unit (ESTU).

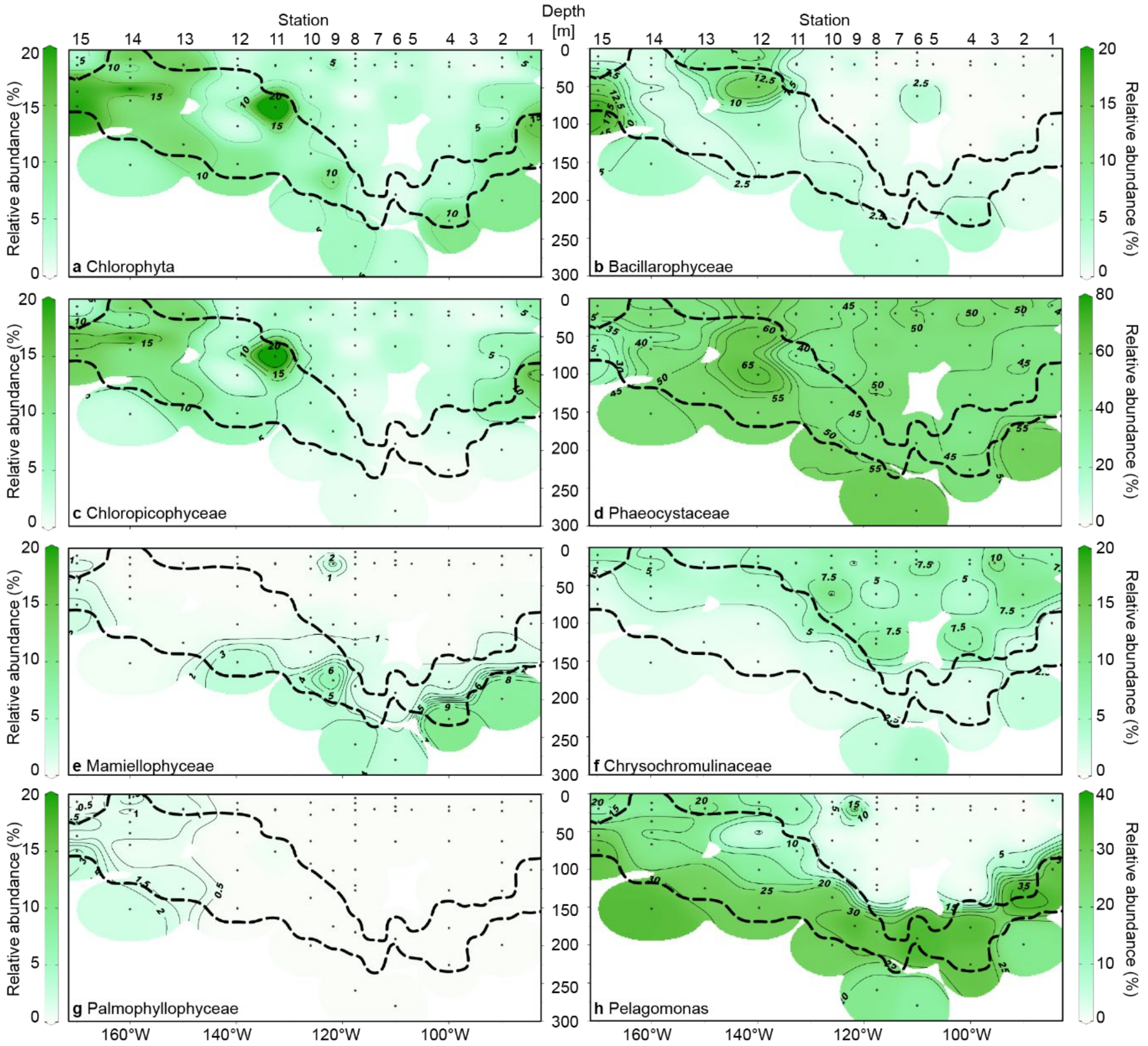

Figure S14: Distribution of plastid 16S rRNA gene relative OTU abundance [%] of total number of chloroplast sequences recovered from each station/depth (rarefied to 400 reads per sample) of (a) Chlorophyta, (b) Bacillariophyceae (diatoms) (c) Chloropicophyceae, (d) Phaeocystaceae, (e) Mamiellophyceae, (f) Chrysochromulinaceae, (g) Palmophyllophyceae and (h) *Pelagomonas* of the upper 300 m of the South Pacific Gyre. Black dots mark the individual samples. The thick dashed line represents the 0.5 µg l^{-1} chl *a* isoline and illustrates the chl *a* max.

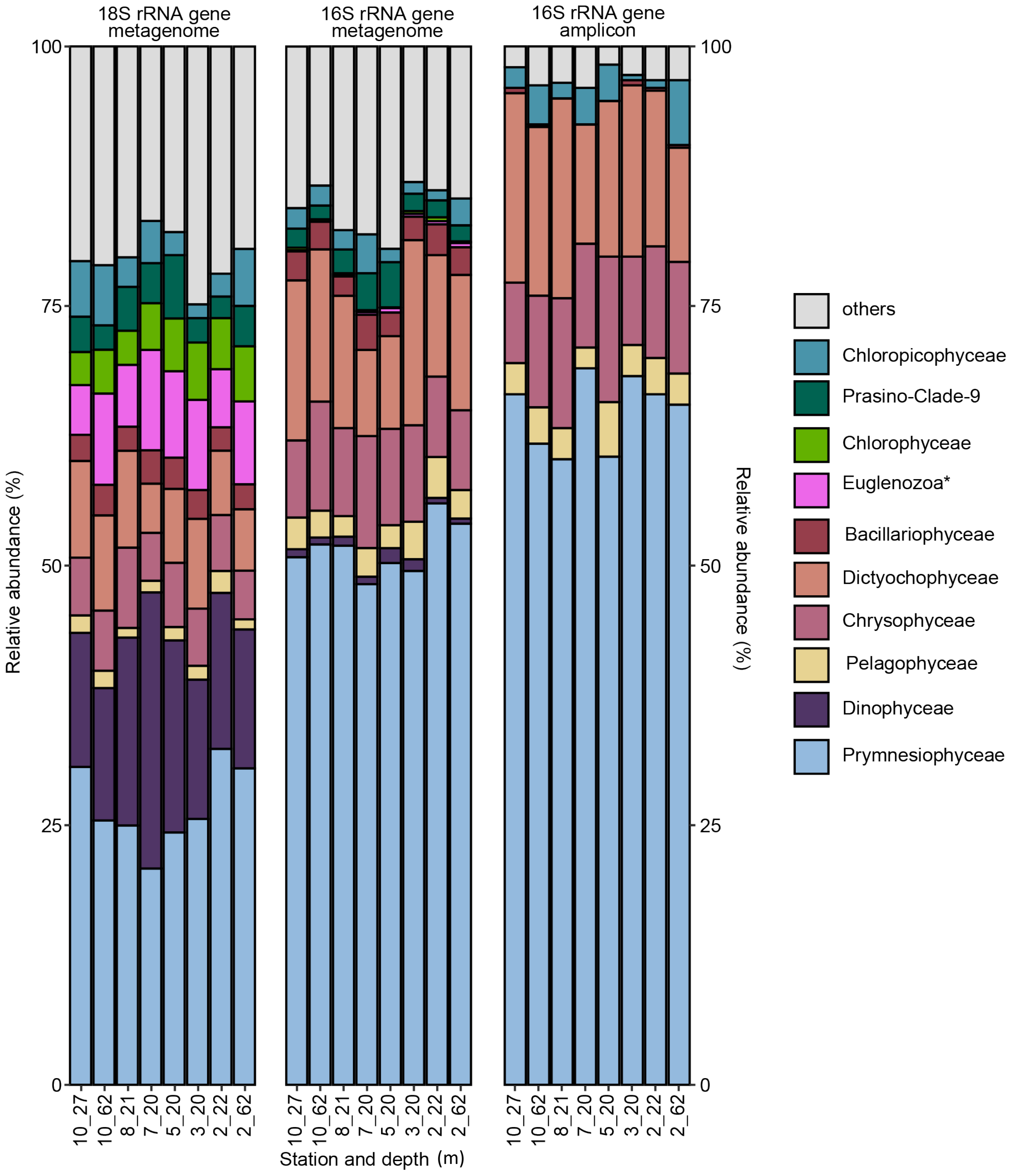

Figure S15: Taxonomic distribution and relative abundance of chloroplast 16S rRNA gene sequences from gene amplicon sequencing compared to shotgun 16S rRNA and photosynthetic 18S rRNA gene sequences derived by metagenomes in eight samples from the euphotic zone of the SPG. Sequences are grouped at the class-level. *Taxonomic affiliation is only valid at phylum level.

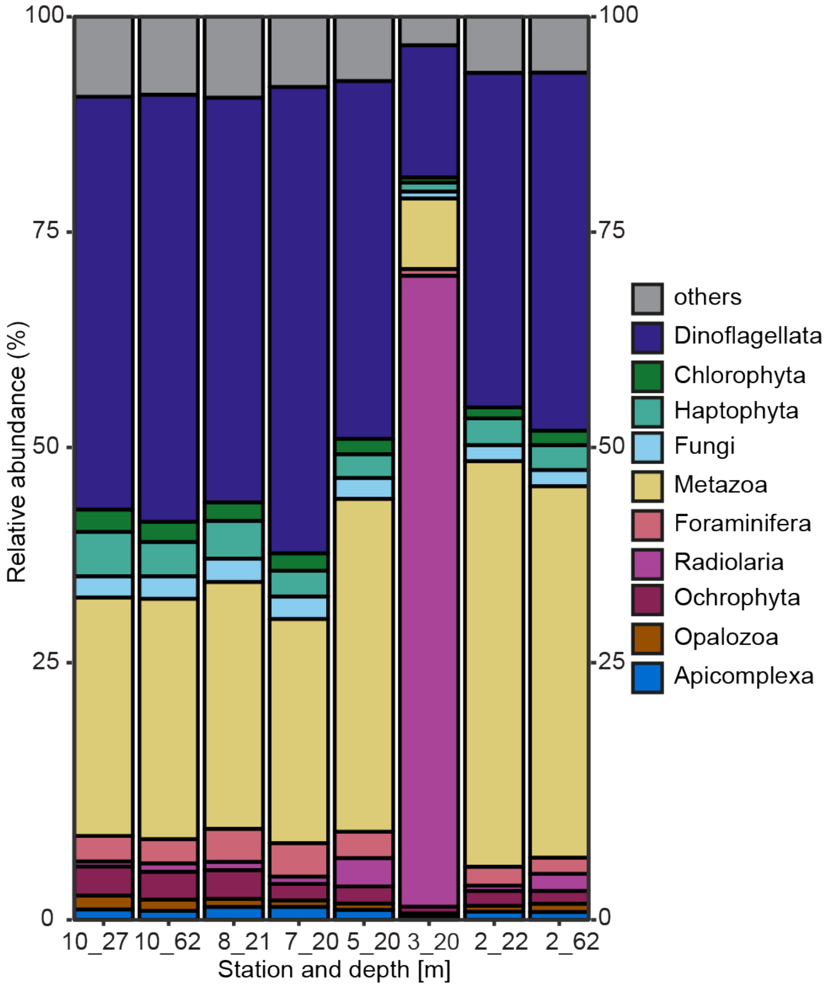

Figure S16: Taxonomic distribution and relative abundance of 18S rRNA gene sequences from shotgun metagenomes in eight samples from the euphotic zone. Sequences are grouped on the phylum level.

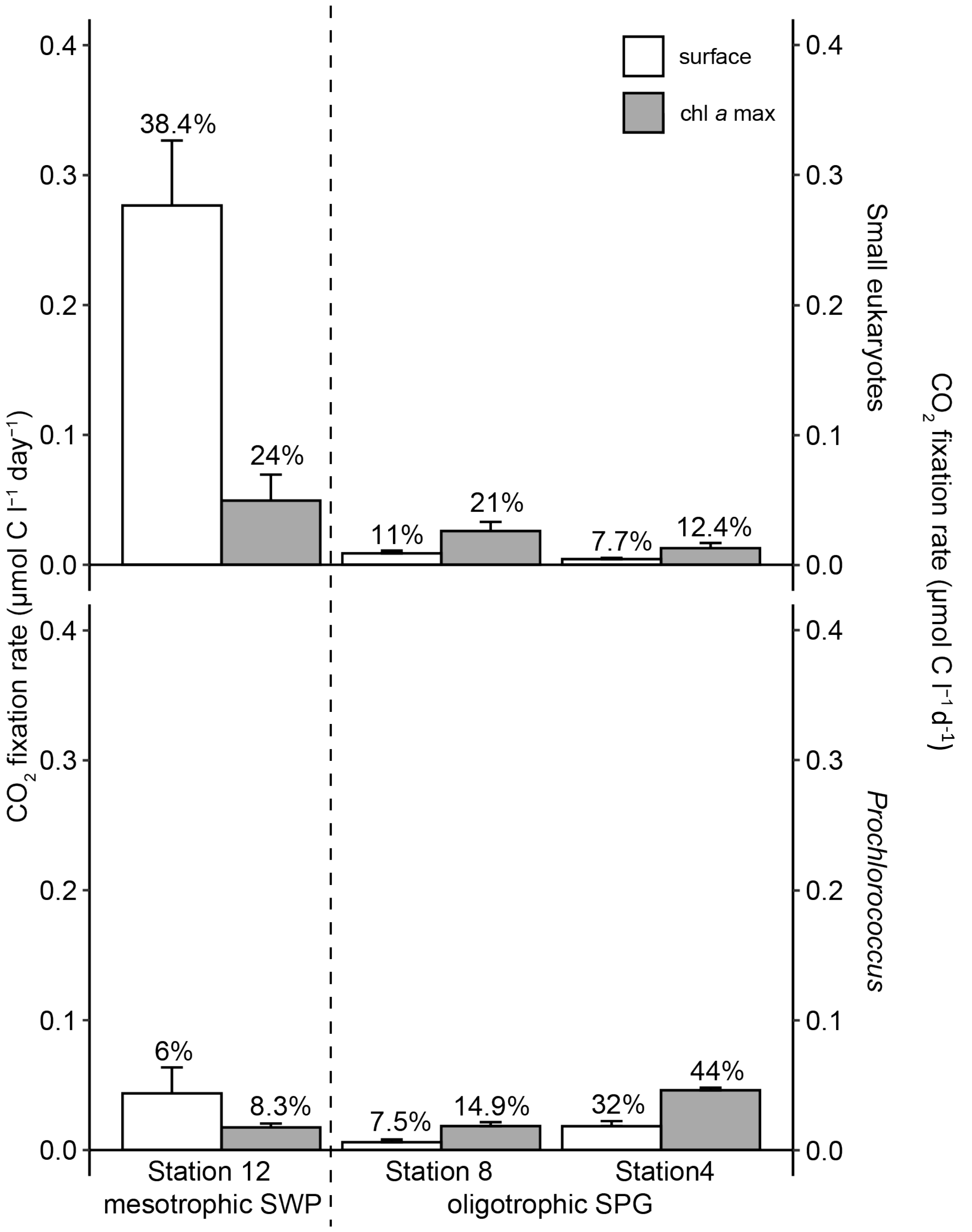

Figure S17: Contributed CO_2 fixation rates for small photosynthetic eukaryotes and for *Prochlorococcus* of the surface (white bars) and chl *a* max (gray bars). The percentages indicate the fraction of volumetric CO_2 fixation by small photosynthetic eukaryotes and *Prochlorococcus* to the bulk CO_2 fixation rate. The error bars indicate the standard error.

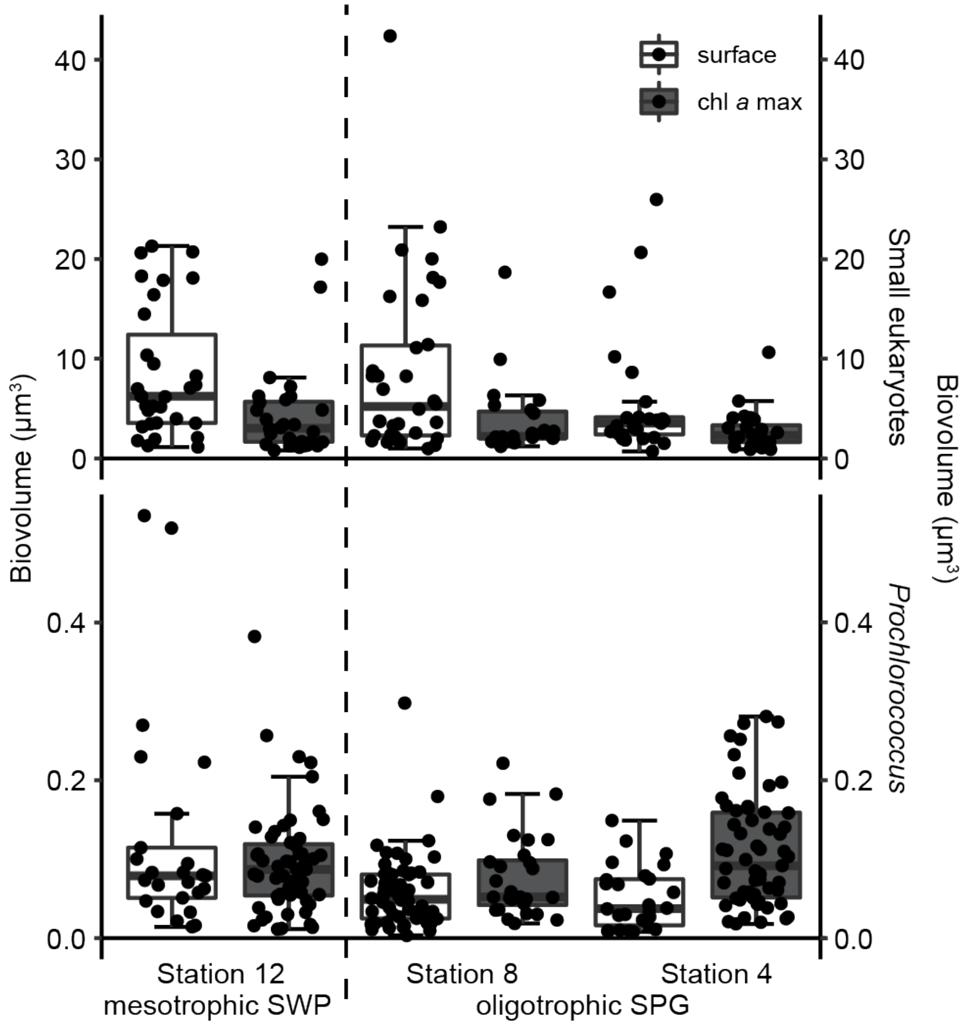

Figure S18: Biovolume [µm^3] of measured small photosynthetic eukaryotes and *Prochlorococcus* in the surface waters (white) and chl *a* max (gray) of the South Pacific Gyre. Black dots indicate measurements of cells above the median growth rate, while the gray dots present measurements of cells below the median growth rate. For each boxplot: dark horizontal line indicates the median, the box boundaries span the 1st (25th percentile) to the 3rd quartile (75th percentile), and the whiskers encompass data points within 1.5 x interquartile range of the selected measurements.

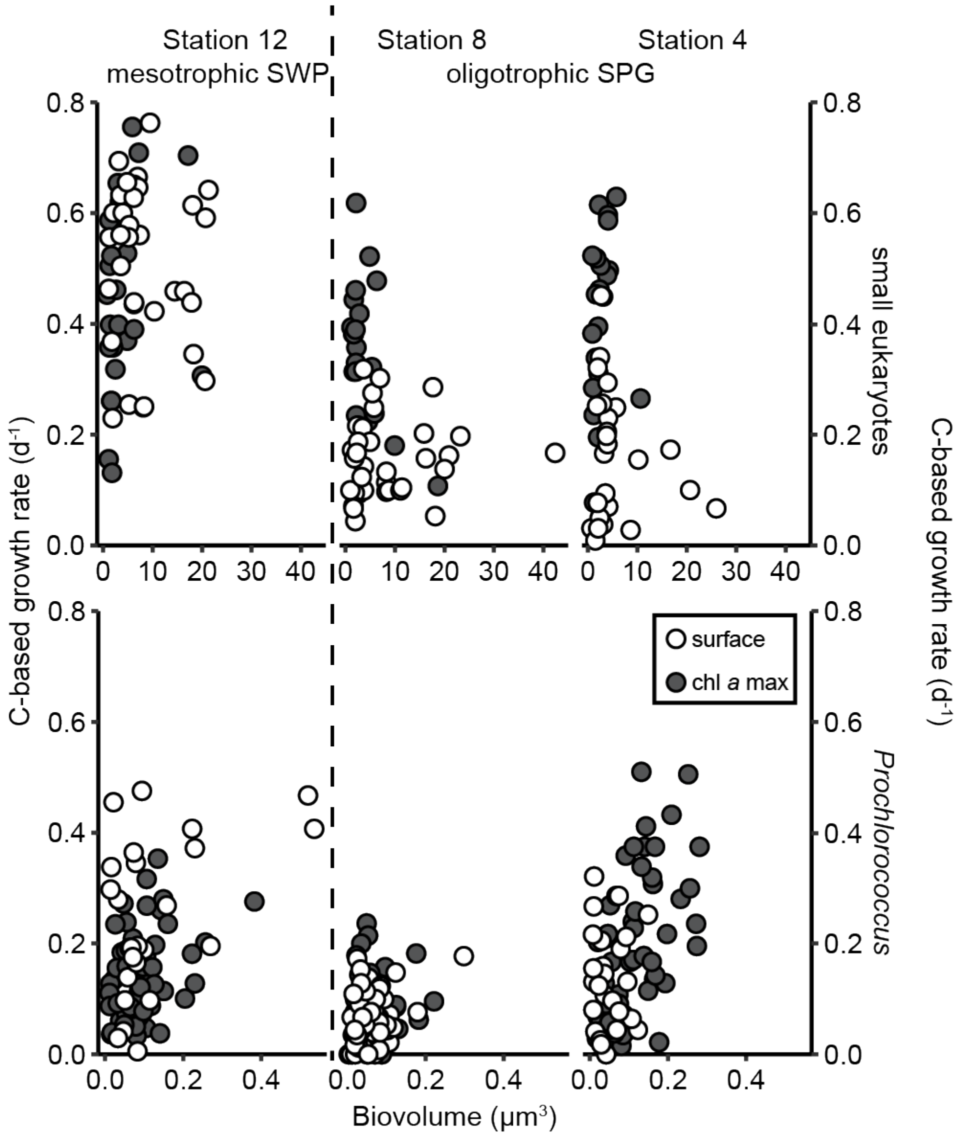

Figure S19: C-based growth rate as a function of biovolume for small photosynthetic eukaryotes (upper panel) and *Prochlorococcus* (lower panel).

III. Extended Description of Methods & Materials

Sampling, hydrography and underwater light field

Sampling was carried out during the *RV Sonne* “UltraPac” cruise (SO245) from Antofagasta, Chile (17 December2015) to Wellington, New Zealand (28 January 2016) (4) (See Station List S2). Temperature, salinity, oxygen and density were examined using a CTD (Sea-Bird Scientific SBE 911plus probe) attached to a Carousel Water Sampler (SBE 32) containing 24x12-liter bottles(5). The system was equipped with double temperature (SBE 3), conductivity probes (SBE 4), a pressure sensor (Digiquartz), an oxygen sensor (SBE 43), an altimeter (Teledyne Benthos) and a chlorophyll fluoromter combined with a turbidity sensor (Sea-Bird Scientific ECO-AFL/FL). Data were recorded with the Seasave V/.23.1 software and processed using Sea-Bird SBE Data Processing software. The ship position was derived from the shipboard GPS-system linked to the CTD data. Sensors were pre-calibrated by the manufactures. Salinity was quality checked by reference samples (n=30), measured with a Precision Salinometer (Optimare OPS S/N 004) five months after the cruise.

A HyperPro II profiling system (SeaBird Scientific, former Satlantic) was used to collect hyperspectral underwater light field data in free-falling mode. The system consisted of one hyperspectral irradiance and one hyperspectral radiance sensor. A second hyperspectral irradiance sensor was mounted on the research vessel for matching above-water irradiance reference measurements. Profiles were conducted at each station depending on sea and weather conditions, with deployments at 50 m away from the ship to avoid ship shadow. All sensors were pre-calibrated by the manufacturer and validated prior to the cruise with a reference lamp. Data were recorded with the SatView software (V 2.9.5_7), pre-processed from raw to Level 3a using the ProSoft Processing software (V 7.7.19_2) and binned in 1 m depth intervals. A dark correction was made automatically based on shutter measurements by the instrument. Post-processing was made in accordance with (6, 7). The photosynthetic active radiation (PAR) was integrated from 400 to 700 nm, considering the onboard reference measurements. The spectral types of 1% downwelling irradiance (Ed) were determined to compare penetration depth and wavelength bands for each station.

Nutrients, Chlorophyll a and Dissolved Organic Carbon and Nitrogen

Dissolved inorganic phosphorus (PO_4^{3-}), nitrite (NO_2^-) and nitrate (NO_3^-) (and silicate (Si)) concentrations were measured with the QuAAtro39 autoanalyzer (Seal Analytical) according to the methods of Strickland and Parsons(8, 9). Seawater low-nutrient standards (OSIL) were used as a secondary standard to test primary standard calibrations. Low concentrations of dissolved phosphate (<300 nM) were determined with a long waveguide capillary cell (LWCC) set-up, which included a Traacs 800 pump and autosampler, a 100 cm LWCC (World Precision Instruments), a HL 2000 Lamp (Ocean Optics) and a STS-VIS Miniature Spectrophotometer (Ocean Optics).

Chlorophyll *a* (chl *a*) samples were filtered (1 L) onto 25 mm GF/F filters (Whatman) with a pore size of 0.7 µm and stored in 10 mL polypropylene tubes at -20 °C in the dark until further processing. Chl *a* extraction and measurements were performed on board after (10, 11). Chl *a* was extracted in 90% acetone and concentrations were measured fluorometrically using a standard curve with 8 chl *a* standard dilutions (Sigma-Aldrich; 4.35 $\mu g\ l^{-1}$ to 198.3 $\mu g\ l^{-1}$) that were verified spectro-photometrically (Thermo Scientific Genesys 10S UV-Vis Spectrometer, Waltham, (12). Fluorescence measurements were blank-corrected with 90% acetone.

CO_2 fixation experiments

Rates of CO_2 fixation, were determined at six depths over the upper water column (< 300 m) at stations, 1 (only two depths), 2, 4, 6, 8, 10, 12, 14, and 15. CO_2 fixation rates were determined through a 24-hours-incubation of collected seawater with the addition of dissolved inorganic ^{13}C ($DI^{13}C$). The ^{13}C method applied in this study is comparable in approach, and for oligotrophic regions, comparable in sensitivity to the classical ^{14}C method (13). Analogous to the $^{14}CO_2$ tracer method, which after more than a half-century of use and refinement is the basis for calibrating algorithms used to estimate surface productivity from satellite data (14), the $^{13}CO_2$ method yields rates lying between gross primary productivity (GPP) and net primary productivity (NPP) (14-16) and references therein). The difference between GPP and NPP depends on the rate of oxidation of fixed C back to CO_2 via photorespiration and mitochondrial respiration by the CO_2-fixing phototrophs during the course of the experiment. As net primary productivity is the measure of CO_2 uptake that we wish to constrain, incubation times are set to 24 hours in order to allow phototrophic mitochondrial respiration to run its full course. As we

are also interested in obtaining near instantaneous CO_2 fixation rates, incubation times are as short as possible, thus limited to one 24-hour incubation cycle.

Incubations were performed in on-deck incubators that were kept at surface water temperature via seawater flow-through and adjusted for three light levels in order to bracket the light conditions experienced by phytoplanktion within the water column. High-light, photo-inhibition effects can lead to an underestimation of CO_2 fixation rate. Conversely, under-illumination may lead to an overestimation, as isotope labeling methods ^{14}C and ^{13}C produce only positive fixation rates and cellular respiration is not registered (see above and e.g. Marra 2009 (ref. 14)). We sampled and transported samples to and from the incubators under darkened conditions. Incubations were adjusted to three different light levels using blue filters (724 Ocean Blue for upper two depths, 132 special medium blue for the middle two depths and 071 Tokyo Blue for the lower two depths, Lee Filters) to simulate the optical characteristics as obtained from the light spectral intensity profiles from Station 1 (Fig. S1b). Incident radiation at the surface can vary up to two-fold. For instance, during the noontime PAR measurements on January 8, shipboard measurements of incident global radiation were 400 W/m^2, but reached values of up to 680 W/m^2 during the course of the day. As shown in Figure S2a, PAR intensities within the euphotic zone at midday can vary more nearly two-fold. The problem of performing incubations with ^{14}C at fixed depths (i.e. no vertical transport through varying light fields) as a function of mixed layer depths, light attenuation and vertical diffusivities has been considered (17). For open ocean regions, they found that incubation derived CO_2 fixation rates values accurately represented in situ rates for a freely mixed euphotic zone (17). Decades of evaluation have shown that when performed with care ^{14}C and ^{13}C incubation methods provide robust estimates of CO_2 fixation into biomass in the euphotic zone, i.e., net primary productivity.

Incubations were performed in triplicates with the addition of $DI^{13}C$ ($NaH^{13}CO_3$, ≥98% Sigma-Aldrich) to ~5% $DI^{13}C$. The natural abundance of ^{13}C-CO_2 in extant particulate organic carbon (POC) was determined from untreated controls that were incubated together with the triplicate experimental bottles. After 24 hours, samples were filtered (under low vacuum; with a membrane pump Liquiport (KNF Neuberger) with constant vacuum of 300 mbar (700 mbar absolute)) on pre-combusted (6 hours at 450 °C) 25 mm GF/F filters (Whatman) and stored at -20 °C until further analysis. Subsamples (100-200 ml) for nanoscale-secondary ion mass spectrometry (nanoSIMS) were taken from the ^{13}C incubation water, fixed with 1% (w/v)

paraformaldehyde for 2 hours at room temperature or overnight at 4 °C (12-24 hours), filtered onto gold sputtered (40 nm) polycarbonate filters (type GTTP; pore size: 0.2 µm; diameter: 25 mm; Millipore), and stored at -20 °C for later analysis. Final labeling percentage of ~5 at% ^{13}C in the DIC pool (final) was measured at the end of the incubation on board by membrane inlet mass spectrometry (MIMS; GAM200, IPI) using a salt-ice mixture as a water trap.

GF/F filters were dried for ~1 hour at 60 °C, acidified overnight over fuming HCl (37%, Merck), re-dried for ~2 hours at 60 °C and pelletized in tin cups. Samples were analyzed for particulate organic carbon and nitrogen (POC and PON) content and isotopic composition using an elemental analyzer (Thermo Flash EA, 1112 Series) coupled to a continuous-flow isotope ratio mass spectrometer (Delta Plus XP IRMS; Thermo Finnigan). IAEA standards N1, N2, USGC40, and CH6 and CH7 were used to calibrate the isotope ratio output of the instrument. Additionally, caffeine standards were measured every 6 samples, in order to evaluate precision and drift, as well as providing standards for absolute C and N content.

CO_2 fixation rates were calculated from the incorporation of $DI^{13}C$ into biomass using the following equation (18).

Equation 1 $$CO_{2-fix\,rate} = \frac{\left(A\%^{POC}_{sample} - A\%^{POC}_{NA\%_{control}}\right)}{\left(A\%_{DIC} - A\%^{POC}_{NA\%_{control}}\right)} \times \frac{[PC]}{\Delta t}$$

where $A\%^{PC}_{sample}$ is the atom % ^{13}C in particulate organic carbon (POC); $A\%_{DIC}$ is the atom percent of $DI^{13}C$ in the total DIC pool; and $A\%^{PC}_{NA\%_{control}}$ is the natural abundance of particulate C (atom %) in the control incubations that were simultaneously incubated with the other bottles with no added $DI^{13}C$. At every IRMS run a pre-combusted GF/F filter was measured as a blank and samples were blank-corrected. Samples with POC/PON concentrations that were estimated to be too low to be precisely quantified by the EA-IRMS were spiked with 89.1 nmol of caffeine (10 µl solution). Twenty-seven spiked blank filters (also with 89.1 nmol caffeine) were used to mass-balance POC and the atom% ^{13}C of the samples treated with the caffeine solution. The caffeine-spiked filters were also used to establish the detection limits for each individual incubation. The necessary threshold for enrichment was defined as three times the standard

deviation of the atom% ^{13}C (0.0145 atom% ^{13}C) of the caffeine-spiked GF/F filters plus the natural abundance of the sample (i.e. the time-zero point).

Areal rates in the upper mixed layer were estimated by approximating the distribution of volumetric rates versus depths of the upper mixed layer with trapezoids. The rate at the surface (z=0) was assumed to be the same as the surface (e.g. 10 meters). Volumetric rates were assumed constant throughout the deep chl *a* max. The thickness of the deep chl *a* max was determined from peak chl *a* values (>0.4 ug chl *a* l^{-1})

Cell enumeration of Prochlorococcus and small photosynthetic eukaryotes

Cell numbers for *Prochlorococcus*, *Synechococcus* and small photosynthetic eukaryotes (1-5 µm) were obtained by an Accuri C6 (BD Biosciences) flow cytometry equipped with a blue laser (488 nm). Samples were run immediately upon collection of water samples from the CTD. Unstained cells were analyzed first by collecting signals for 5 minutes at a rate of 66 µl min^{-1} and by gating on forward scatter (FSC) and chl fluorescence (FL3). Nano- and pico-eukaryotes were distinguished by size using FSC (Fig. S3). Cell size for flow cytometry analysis was estimated by comparison to micron (Cat 833) and sub-micron (Cat 832) bead calibration kits (Bang Laboratories). Cell numbers in all treatments were calculated by the use of AccuCount Ultra Rainbow Fluorescent Particles (Spherotech). Day to day instrument performance was assessed using 6 peak and 8 peak validation beads (Spherotech).

Accurate determinations of *Prochlorococcus* in high light intensity, oligotrophic environments such as the SPG are known to be highly problematic (19). *Prochlorococcus* populations with dim chlorophyll fluorescence were identified by gating on green fluorescence (FL1) and chl *a* fluorescence (FL3) after staining with SYBR Green (Molecular Probes S7585) (Figures S4-S8). *Prochlorococcus* cell numbers were subsequently cross-checked with direct cell counts and onboard contemporaneous DNA sequence reads (2) (Figure S12). Heterotrophic prokaryotes were determined in subsamples that had been stained with SYBR Green I Nucleic Acid Stain (Biosciences) for at least 15 minutes in the dark at room temperature.

Single-cell sample imaging

Catalyzed reporter deposition-fluorescent in situ hybridization (CARD-FISH) was used to identify *Prochlorococcus* cells according to (20). Filters were punched out into 5 mm circles

prior to the CARD-FISH procedure, bleached for 10 minutes with 0.01 M HCl at room-temperature, and washed with 1x phosphate buffered saline (PBS) buffer. Bacterial cells were made permeable by incubating the filter pieces in lysozyme (10 mg ml^{-1}) for 45 minutes at 37 °C. Samples were washed in deionized water before performing hybridization. CARD-FISH was performed using 5'-HRP-labeled oligo nucleotide probes (PRO405; AGAGGCCTTCGTCCCTCA, sequence (5' - 3')) (ref. 21). Filter pieces were incubated with the PRO405 probe in 15% formamide containing hybridization buffer for 2-3 hours at 46 °C. Filters were washed in pre-warmed washing buffer containing NaCl (0.03 M final concentration), 20 mM Tris-HCl (pH 7.5), and 0.01% SDS for 15min at 48 °C and then washed for 10 min in 1xPBS at room temperature. Filter pieces were incubated for 45 min at 46 °C in the dark in amplification buffer containing 0.15% H_2O_2 and 20 µg Oregon Green 488 labelled tyramides (Molecular Probes). Filter pieces were washed 15 min in the dark with 1xPBS and dried. Cells were counter stained with 4'6-diamidino-2-phenylindol (DAPI, 1 mg ml^{-1}) for 10 min at 4°C in the dark. Filter pieces were washed in deionized water dried and mounted on a microscope slide. Positively hybridized *Prochlorococcus* cells and small photosynthetic eukaryotic cells (identified by chloroplast autofluorescence) were marked using a laser micro-dissection microscope (DM 6000 B, Leica Microsystems). Filter pieces were embedded in a Citifluor/Vectachield mixture and epifluorescence images of the marked fields of view were obtained with a Zeiss Axiocam 506 mono or Axiocam MRm camera (Zeiss) for orientation during nanoSIMS analysis.

Isotopic compositions of marked cells were determined with a CAMECA nanoSIMS 50L ion microprobe (CAMECA, Gennevilliers, France) at two depths (~20 m and chl *a* max) at station 4, 8 and 12. Secondary ion images of $^{12}C^-$, $^{13}C^-$, $^{19}F^-$, $^{12}C^{14}N^-$, $^{12}C^{15}N^-$, $^{31}P^-$ and $^{32}S^-$ were recorded simultaneously on 7 electron multiplier detectors. All samples were pre-sputtered with a Cs+ primary ion beam with a current of ~300 pA for 20-120 sec, depending on the target organism. Measurements were performed with a raster size of 10x10 to 30-30 µm, with a dwelling time of 1 ms per pixel and a 256x256 pixel resolution over minimum of 40 planes. The data was processed using Look@NanoSIMS software (22). For every measurement run the recorded secondary ion images of at least 10 of the measured planes were drift corrected and accumulated. Region of interests (ROIs) around cell structures were defined using the RGB combined image of $^{12}C^{14}N^-$ and $^{32}S^-$. For each ROI the $^{13}C/^{12}C$ ratios were calculated. The variability of all background values (average ± 3xSD; 1.13 atom %) of the dataset was used to

estimate the detection limit for significant isotope enrichments. Poisson error across all planes was <5%. The natural abundance (NA= 1.1 atom %) of ^{13}C atom % was used from the EA-IRMS measurements (average from the respective samples measured with the nanoSIMS).

Single cell calculations

The cell size, as determined with Look@NanoSIMS software (22), was used to calculate biovolume (BV). For size determination of cells, ROIs were drawn after the first two planes were accumulated, as we have noticed that during the measurement that cell size is sometimes reduced as the beam removes parts of the cell.

Prochlorococcus and photosynthetic small eukaryotes were considered as a prolate spheroid and thus the following equation was used for BV calculations:

Equation 2 $$BV = \frac{1}{6}\pi \times b \times 2 \times a$$

where b is the width and a is the length of the cell.

The carbon (C) per cell was calculated based on the BV with the formula by (23) for small eukaryotes

Equation 3 $$pg\ C\ cell^{-1} = 0.433 \times BV^{0.863}$$

and by (24) for *Prochlorococcus*

Equation 4 $$fg\ C\ cell^{-1} = 197 \times BV^{0.46}.$$

Growth rate were estimated based on the incorporations of $DI^{13}C$ into biomass assuming exponential growth and an even distribution of the isotopes in the biomass during cell division:

Equation 5 $$C_{based}GrowthRate\ [d^{-1}] = log_2 \left[\frac{A\%_{DIC}}{A\%_{xs-DIC} - A\%_{xs-cell}}\right] \times \frac{1}{t}$$

where $A\%_{DIC}$ is the atom % excess over background in the DIC pool; $A\%_{xs-cell}$ is the atom % excess over background measured in the photosynthetic small eukaryote and *Prochlorococcus* cells; and t is incubation time in days.

Contributions of small photosynthetic eukaryotes and *Prochlorococcus* to the bulk CO_2 fixation were calculated with the median single-cell CO_2 fixation rates of the respected group of organisms, station and depth multiplied by the abundance. For the rates measured at 20 m depth abundance the respective data taken from the surface to 40 m were used and for the chl *a* max abundance data within the 0.5 µg chl a l^{-1} isoline were used.

DNA extraction and 16S rRNA metabarcoding

Two liters of seawater were filtered onto polyvinylidene fluoride membrane filters (0.22 pore size µm; 47 mm diameter, Millipore), frozen immediately and stored at -80 °C. DNA was extracted using the Qiagen RNA/DNA Mini-Kit, after crushing the filter under liquid N_2 with a sterile pipette tip. Prior to adding the kit lysis buffer, we added 200 µl of lysozyme (5 mg ml^{-1}), incubated for 10 minutes at room temperature, and transferred the lysate to the QiaShredder column. DNA was finally eluted with 60 µl Qiagen DNA buffer.

Partial 16S rRNA genes were amplified using previously described primers targeting the V4-V5 variable region of the 16S rRNA gene (forward: GTGYCAGCMGCCGCGGTAA; reverse: AAACTYAAAKRAATTGRCGG) (25,26). Tag sequencing of DNA gene amplicons was carried out on an Illumina MiSeq instrument using 2 x 300 bp paired-end v3 chemistry at the Integrated Microbiome Resource, Dalhousie University (27). Further details on the scripts and codes for the pipeline can be found at http://imr.bio/protocols.html. The 16S rRNA gene amplicon sequence reads were processed using a QIIME1-based workflow (https://github.com/LangilleLab/microbiome_helper/wiki/16S-Bacteria-and-Archaea-Standard-Operating-Procedure) (28) available from the Microbiome Helper repository (27, 29). Briefly, paired-end reads were merged using PEAR (30) and sequences <400 bp or with quality <30 over 90% of bases were discarded. Chimeras were removed using VSEARCH (31). The programs SortMeRNA (32) and Sumaclust (33) were used for picking operational taxonomic units (OTUs) (defined by 97% sequence similarity) utilizing Greengenes (16S rRNA) as a reference database (34). Sequencing bleed-through was minimized by removing OTUs with a relative abundance of < 0.1%. Gene amplicon reads that mapped to Chloroplasts in the Greengenes database were then extracted and annotated using the updated PhytoREF chloroplast database in accordance of the PR2 version 4.12.0 database (35, 36) as a reference. Reads were rarefied to 400 reads for all further analyses, and seven of 71 original samples with < 400 reads

mapped to chloroplasts were discarded. All 618 chloroplast OTUs sequences obtained were also identified as chloroplasts when cross-checking against the SILVA SSU database (version 138) (ref. 35).

Phylogenetic analysis was performed with the ARB software package (37). Selected sequences from the PhytoREF chloroplast database (35) were used as reference sequences and the taxonomic affiliation of reference sequences was updated according to the PR2 version 4.12.0 database (36). *Prochlorococcus* OTUs from this study were aligned with the SINA 1.3.0 aligner (40) in ARB to the 16S rRNA SILVA 138 SSU (release 99) reference database (38). Sequences were aligned in ARB with MAFFT (40) and checked manually. The top 50 OTUs from this study were aligned to the reference sequences. A maximum-likelihood tree based on 66 selected long reference sequences (≥1300 nucleotides, including three cyanobacterial sequences used as an outgroup to root the tree; accession number: HM217059, HM217083, HM217075) was calculated using RAxML 7.7.2 (ref. 41) with a filter by base frequency filter that excluded alignment positions with sequence uncertainty or missing data. An additional 44 partial reference sequences and the top 50 OTUs from this study were added to the tree with the ARB Parsimony function.

16S and18S rRNA gene sequencing retrieved from metagenomes

Shotgun metagenomes were generated for eight samples from the core of the South Pacific Gyre. Library preparation and sequencing were performed at the Max Planck Genome Center Cologne, Germany (https://mpgc.mpipz.mpg.de/home/). Fifteen ng genomic DNA was used for library preparation with NEBNext Ultra II FS DNA Library Prep Kit for Illumina (New England Biolabs). Library preparation included eight cycles of PCR amplification. Quality and quantity were assessed at all steps via capillary electrophoresis (TapeStation, Agilent Technologies) and fluorometry (Qubit, Thermo Fisher Scientific). Sequencing was performed on HiSeq2500 system (Illumina) with 2 x 250 bp paired end reads. Raw reads were quality trimmed using Trimmomatic (42) with the parameters “LEADING:3 TRAILING:3 SLIDINGWINDOW:4:15 MINLEN:36”. To assess phytoplankton community composition in shotgun metagenomes, 16S and 18S rRNA reads were mapped onto reference databases using phyloFlash (43). The PR2 version 4.12.0 database (36) was used for 18S rRNA based analysis. For 16S rRNA a custom database was created comprising all bacterial and archaeal entries of the SILVA SSU database (version 138) (ref. 38) together with the PhytoREF chloroplast 16S rRNA database (35), with updated taxonomy in accordance to the integrated version in the PR2

database (36). We removed non-chloroplast sequences of the 16S rRNA metagenomic data, as well as sequences from the class of Embryophyceae (land plants), which were assumed to be contaminants. To retrieve only photosynthetic 18S rRNA sequence we filtered based on the results of the chloroplast 16S rRNA metagenomic approach.

petB Gene Distributions

In order to assess the distribution of *Prochlorococcus* ecotypes, we mapped the metagenomic reads from the metagenomes described above to a custom database of the petB gene, a high-resolution taxonomic marker for *Prochlorococcus* ecotypes (3). We also extracted reads from additional shotgun metagenomes (44) that were generated from samples collected with in situ pumps (McLane) at three depths (20 m, above the chl *a* max and below the chl *a* max) of stations 4, 6 and 8 in the core of the gyre. DNA was extracted using the Qiagen AllPrep Bacterial DNA/RNA/Protein kit from a 1/16 portion of 142 mm diameter polyethersulfone membranes (two membranes of 0.8 µm pore size stacked on top of each other, with an effective pore size of 0.45 µm). Library preparation and sequencing were also performed at the Max Planck Genome Center Cologne, Germany. The custom-made petB gene database consisted of the *Prochlorococcus* subset (3). Trimmed reads were mapped against the database using bbmap at ≥99% identity. Calculated Reads per Kilobase per 10^6 Mapped Reads (RPKM) of all entries were summed within each environmentally significant taxonomic unit (ESTU).

IV. References Cited in Supplementary Information

1. Osterholz H, Kilgour D, Storey DS, Lavik G, Ferdelman T, Niggemann J, et al. Accumulation of DOC in the South Pacific subtropical gyre from a molecular perspective. *Mar Chem.* 2021:**231**:103955

2. Reintjes G, Tegetmeyer HE, Bürgisser M, Orlić S, Tews I, Zubkov M, et al. On-site analysis of bacterial communities of the ultraoligotrophic South Pacific gyre. *Appl Environ Microbiol*. 2019; **85**: e00184-00119.

3. Farrant GK, Doré H, Cornejo-Castillo FM, Partensky F, Ratin M, Ostrowski M, et al. Delineating ecologically significant taxonomic units from global patterns of marine picocyanobacteria. *PNAS*. 2016; **113**: E3365-E3374.

4. Ferdelman TG. *RV Sonne SO245 Cruise Report / Fahrtbericht, Antofagasta, Chile: 17. December 2015, Wellington, New Zealand: 28. January 2016, SO245 - Ultrapac: Process oriented biogeochemical, microbiological and ecological investigations of the ultraoligotrophic South Pacific gyre*; e-pub DOI: 10.2312/cr_so245, 2016,

5. Zielinski O, Henkel R, Voß D, Ferdelman TG. Physical oceanography during Sonne cruise SO245 (Ultrapac). *PANGAEA*. 2018;. https://doiorg/101594/PANGAEA890394.

6. Mueller JL, Morel A, Frouin R, Davis C, Arnone R, Carder K, et al. *Ocean optics protocols for satellite ocean color sensor validation, revision 4. Volume III: Radiometric measurements and data analysis protocols*. NASA, Goddard Space Flight Center: Maryland, USA, 2003.

7. Organelli E, Claustre H, Bricaud A, Schmechtig C, Poteau A, Xing X, et al. A novel near-real-time quality-control procedure for radiometric profiles measured by Bio-Argo floats: protocols and performances. *J Atmosphere Ocean Tech*. 2016;**33**:937-951.

8. Strickland JDH, Parsons TR. *A practical handbook of seawater analysis*: Fisheries Research Board of Canada Bulletin; 1972.

9. Ferdelman TG, Klockgether G, Downes P, Lavik G. Nutrient data from CTD Nisken bottles from Sonne expedition SO-245 "Ultrapac". *PANGAEA*, 2019. https://doiorg/101594/PANGAEA899228.

10. Arar EJ, Collins GB. Method 445.0: in vitro determination of chlorophyll *a* and pheophytin a in marine and freshwater algae by fluorescence: United States Environmental Protection Agency, Office of Research and Development, National Exposure Research Laboratory; 1997.

11. Welschmeyer N, Naughton S. Improved chlorophyll *a* analysis: single fluorometric measurement with no acidification. *Lake Reserv Manage*. 1994; **9**: 123.

12. Arar E. Determination of chlorophylls a and b and identification of other pigments of interest in marine and reshwater algae using high performance liquid chromatography with visible wavelength detection. Method 447. United States Environmental Protection Agency, Office of Research and Development, National Exposure Research Laboratory. 1997.

13. López-Sandoval DC, Delgado-Huertas A, Agustí S. The ^{13}C method as a robust alternative to ^{14}C-based measurements of primary productivity in the mediterranean sea. *J Plankton Res.* 2018; **40**: 544-554.

14. Marra J. Net and gross productivity: weighing in with ^{14}C. *Aquat Microb Ecol.* 2009; **56**: 123-131.

15. Bender M, Grande K, Johnson K, Marra J, Williams PJL, Sieburth J, et al. A comparison of four methods for determining planktonic community production1. *Limnol Oceanogr.* 1987; **32**:1 085-1098.

16. White AE, Barone B, Letelier RM, Karl DM. Productivity diagnosed from the diel cycle of particulate carbon in the North Pacific subtropical gyre. *Geophys Res Lett*. 2017; **44**: GL071607.

17. Ross ON, Geider RJ, Piera J. Modelling the effect of vertical mixing on bottle incubations for determining in situ phytoplankton dynamics. II. Primary production. *Mar Ecol Prog Ser*. 2011; **435**: 33-45.

18. Großkopf T, Mohr W, Baustian T, Schunck H, Gill D, Kuypers MMM, et al. Doubling of marine dinitrogen-fixation rates based on direct measurements. *Nature*. 2012; **488**: 361-364.

19 Ribeiro CG, Marie D, Santos ALd, Brandini FP, Vaulot D. Estimating microbial populations by flow cytometry: comparison between instruments. *Limnol Oceanogr Methods*. 2016; **14**: 750-758.

20. Pernthaler A, Pernthaler J, Amann R. Fluorescence in situ hybridization and catalyzed reporter deposition for the identification of marine bacteria. *Appl Environ Microbiol*. 2002; **68**: 3094-3101.

21. West NJ, Schönhuber WA, Fuller NJ, Amann RI, Rippka R, Post AF, et al. Closely related *Prochlorococcus* genotypes show remarkably different depth distributions in two oceanic regions as revealed by in situ hybridization using 16S rRNA-targeted oligonucleotides. *Microbiology.* 2001; **147**: 1731-1744.

22. Polerecky L, Adam B, Milucka J, Musat N, Vagner T, Kuypers MMM. Look@NanoSIMS – a tool for the analysis of nanoSIMS data in environmental microbiology. *Environ Microbiol*. 2012; **14**: 1009-1023.

23. Verity PG, Robertson CY, Tronzo CR, Andrews MG, Nelson JR, Sieracki ME. Relationships between cell volume and the carbon and nitrogen content of marine photosynthetic nanoplankton. *Limnol Oceanogr*. 1992; **37**: 1434-1446.

24. Khachikyan A, Milucka J, Littmann S, Ahmerkamp S, Meador T, Könneke M, et al. Direct cell mass measurements expand the role of small microorganisms in nature. *Appl Environ Microbiol*. 2019; **85**: AEM00493-00419..

25. Walters W, Hyde ER, Berg-Lyons D, Ackermann G, Humphrey G, Parada A, et al. Improved bacterial 16S rRNA gene (v4 and v4-5) and fungal internal transcribed spacer marker gene primers for microbial community surveys. *MSystems*. 2016; **1**: e00009-00015.

26. Parada AE, Needham DM, Fuhrman JA. Every base matters: Assessing small subunit rRNA primers for marine microbiomes with mock communities, time series and global field samples. *Environ Microbiol*. 2016; **18**: 1403-1414.

27. Comeau AM, Douglas GM, Langille MG. Microbiome helper: a custom and streamlined workflow for microbiome research. *MSystems*. 2017; **2**: e00127-00116.

28. Caporaso JG, Kuczynski J, Stombaugh J, Bittinger K, Bushman FD, Costello EK, et al. Qiime allows analysis of high-throughput community sequencing data. *Nat Methods.* 2010; **7**: 335.

29. Haas S, Desai DK, LaRoche J, Pawlowicz R, Wallace DW. Geomicrobiology of the carbon, nitrogen and sulphur cycles in Powell Lake: a permanently stratified water column containing ancient seawater. *Environ Microbiol*. 2019;**21**:3927-3952.

30. Zhang J, Kobert K, Flouri T, Stamatakis A. Pear: a fast and accurate Illumina paired-end read merger. *Bioinformatics*. 2013; **30**: 614-620.

31. Rognes T, Flouri T, Nichols B, Quince C, Mahé F. Vsearch: A versatile open source tool for metagenomics. *PeerJ*. 2016; **4**: e2584.

32. Kopylova E, Noé L, Touzet H. Sortmerna: fast and accurate filtering of ribosomal RNAs in metatranscriptomic data. *Bioinformatics*. 2012; **28**: 3211-3217.

33. Mercier C, Boyer F, Bonin A, Coissac E (eds). Sumatra and Sumaclust: fast and exact comparison and clustering of sequences. *SeqBio 2013 Workshop* 2013: (abstract).

34. DeSantis TZ, Hugenholtz P, Larsen N, Rojas M, Brodie EL, Keller K, et al. Greengenes, a chimera-checked 16S rRNA gene database and workbench compatible with ARB. *Appl Environ Microbiol*. 2006; **72**: 5069-5072.

35. Decelle J, Romac S, Stern RF, Bendif EM, Zingone A, Audic S, et al. PhytoREF: A reference database of the plastidial 16S rRNA gene of photosynthetic eukaryotes with curated taxonomy. *Molec Ecol Res*. 2015;**15**:1435-1445.

36. Guillou L, Bachar D, Audic S, Bass D, Berney C, Bittner L, et al. The protist ribosomal reference database (PR2): A catalog of unicellular eukaryote small sub-unit rRNA sequences with curated taxonomy. *Nucleic Acids Res*. 2012; **41**: D597-D604.

37. Ludwig W, Strunk O, Westram R, Richter L, Meier H, Yadhukumar, et al. ARB: a software environment for sequence data. *Nucleic Acids Res*. 2004; **32**:1363-1371.

38. Quast C, Pruesse E, Yilmaz P, Gerken J, Schweer T, Yarza P, et al. The SILVA ribosomal RNA gene database project: improved data processing and web-based tools. *Nucleic Acids Res.* 2012; 41: D590-D596.

39. Pruesse E, Peplies J, Glöckner FO. Sina: Accurate high-throughput multiple sequence alignment of ribosomal RNA genes. *Bioinformatics*. 2012;**28**: 1823-1829.

40. Katoh K, Misawa K, Kuma Ki, Miyata T. MAFFT: A novel method for rapid multiple sequence alignment based on fast fourier transform*.* *Nucleic Acids Res*. 2002; **30**: 3059-3066.

41. Stamatakis A. Raxml-vi-hpc: maximum likelihood-based phylogenetic analyses with thousands of taxa and mixed models. *Bioinformatics*. 2006; **22**: 2688-2690.

42. Bolger AM, Lohse M, Usadel B. Trimmomatic: A flexible trimmer for illumina sequence data**.** *Bioinformatics.* 2014; **30**: 2114-2120.

43. Gruber-Vodicka HR, Seah BK, Pruesse E. Phyloflash: rapid small-subunit rRNA profiling and targeted assembly from metagenomes. *Msystems*. 2020; **5:** e00920-20.

44. Oggerin de Orube M, Fuchs BM. Unpublished shotgun metagenomes collected from in situ pump samples during R/V Sonne expedition SO245. 2021
